# GDF15 in Liver Fibrosis: Molecular Mechanisms, Immunoregulatory Functions, and Therapeutic Potential

**DOI:** 10.3390/biom16071060

**Published:** 2026-07-20

**Authors:** Xinyun Gan, Longze Zhang, Ting Yu, Sikan Jin, Yan Wu, Rui Xu, Yaqi Zhang, Jidong Zhang, Lin Xu, Xianyao Wang

**Affiliations:** 1 Department of Immunology, Zunyi Medical University, Zunyi 563000, China; 15700676213@163.com (X.G.); 18185861176@163.com (T.Y.); jkk24sa@163.com (S.J.); 13385123453@163.com (Y.W.); 15329199688@163.com (R.X.); 18385381333@163.com (Y.Z.); jidongzhang@zmu.edu.cn (J.Z.); 2 Key Laboratory of Cancer Prevention and Treatment of Guizhou Province, Zunyi Medical University, Zunyi 563000, China; 3 Scientific Research Center, The First People’s Hospital of Zunyi (The Third Affiliated Hospital of Zunyi Medical University), Zunyi 563000, China; zlongze@163.com; 4 Department of Nuclear Medicine, The Affiliated Hospital of Zunyi Medical University, Zunyi 563003, China

**Keywords:** GDF15, liver fibrosis, immunomodulatory, HSCs, target therapy

## Abstract

Liver fibrosis is a chronic pathological process driven by the activation of hepatic stellate cell (HSC) and characterized by the excessive deposition of extracellular matrix (ECM) components in response to persistent liver injury. This condition can lead to progressive hepatic dysfunction, cirrhosis, and ultimately liver failure. Growth differentiation factor 15 (GDF15) has emerged as a pivotal regulator in the initiation and progression of liver fibrosis, exhibiting context-dependent profibrotic and antifibrotic effects. GDF15 modulates multiple cellular processes, including HSC activation and macrophage polarization, as well as the functions of T cells, natural killer cells, B cells and mesenchymal stem cells. This review provides a comprehensive overview of the role of GDF15 in regulating HSC activation and immune cell responses and elaborates on its immunomodulatory functions in attenuating liver fibrosis. Furthermore, we discuss the therapeutic potential of targeting GDF15 for the treatment of liver fibrosis. Ultimately, this review aims to provide a theoretical foundation and propose novel intervention strategies for the early diagnosis and targeted therapy of liver fibrosis.

## 1. Introduction

Liver fibrosis is a reparative pathological process that occurs in response to chronic liver injury and is characterized by the excessive deposition of extracellular matrix (ECM) components [1]. Common triggers, including viral infections, exposure to chemical toxins, and inherited metabolic disorders, lead to the aberrant activation of hepatic stellate cells (HSCs) and other fibrogenic cell populations [2]. Sustained HSC activation is widely recognized as a central driver of fibrogenesis, promoting progressive ECM accumulation that can ultimately result in cirrhosis, hepatocellular carcinoma (HCC), and severe complications such as hepatic encephalopathy and liver failure [3,4,5]. Liver fibrosis poses a significant clinical burden, yet it often progresses insidiously without distinct early symptoms, leading to frequent diagnostic delays [6]. Although evidence suggests potential reversibility under certain conditions, recent FDA approvals, such as semaglutide in August 2025 and resmetirom in March 2024 for patients with metabolic dysfunction-associated steatohepatitis (MASH) and moderate to advanced fibrosis, highlight emerging therapeutic opportunities [7,8]. Nonetheless, a deeper understanding of the molecular mechanisms driving fibrosis progression remains essential for developing more effective preventive and targeted therapies [9,10]. Therefore, identifying early diagnostic biomarkers and advancing targeted treatment strategies continue to be crucial for improving patient outcomes.

Growth differentiation factor 15 (GDF15), also known as macrophage inhibitory cytokine 1 (MIC-1), NSAID-activated gene 1 protein (NAG-1), or placental TGF-Beta (PTGFB), is a divergent member of the transforming growth factor-β (TGF-β) superfamily [11,12,13]. It shares a conserved cysteine-knot structural motif and is widely expressed and secreted by multiple tissues, including the liver, kidney, adipose tissue, skeletal muscle, and pancreatic islets. GDF15 plays a crucial role in maintaining physiological homeostasis and exerts potent regulatory effects on diverse processes such as inflammation, energy metabolism, cardiovascular function, obesity, tumor progression, cachexia and aging [14,15,16,17,18]. Under normal conditions, GDF15 expression is tightly regulated but can be markedly upregulated in response to tissue injury, cellular stress, inflammation, mitochondrial dysfunction, nutrient deprivation, or hormonal fluctuations [14]. The primary site of GDF15 action is the hindbrain, specifically the nucleus tractus solitarius within the medulla oblongata, where it binds to the glial cell line-derived neurotrophic factor receptor alpha-like (GFRAL)–RET heterodimeric receptor complex [19]. Notably, GFRAL expression is confined to the brainstem and does not interact structurally with canonical TGF-β ligands or members of the glial cell line-derived neurotrophic factor family [20]. Upon ligand–receptor binding, GDF15 regulates intracellular signaling pathways, most prominently the extracellular signal-regulated kinase (ERK) and AKT/protein kinase B cascades, thereby modulating downstream physiological responses [21,22]. Functioning predominantly as a circulating hormone, GDF15 is released into the bloodstream by stressed peripheral cells and targets the area postrema and nucleus tractus solitarius [23,24]. These regions are circumventricular organs that lack a functional blood–brain barrier, thereby permitting direct access of GDF15 to neuronal GFRAL–RET complexes [25]. Through this central signaling axis, systemic effects including appetite suppression and metabolic adaptation are mediated [23]. These pleiotropic functions underscore its significance as both a biomarker and a potential therapeutic target.

Recent studies have highlighted the critical involvement of GDF15 in the pathogenesis of liver diseases, particularly liver fibrosis [26]. During the early stages of fibrosis, GDF15 is thought to confer protective effects by modulating immune responses and mitigating oxidative stress [26]. However, as fibrosis advances, GDF15 expression becomes markedly elevated and correlates positively with disease severity [27]. Although GDF15 appears hepatoprotective during acute injury, its sustained elevation in chronic or advanced fibrotic states may contribute to maladaptive tissue remodeling. Consequently, elevated circulating GDF15 has been proposed as a potential biomarker for advanced fibrosis and a predictor of HCC. Further mechanistic studies are therefore required to elucidate this stage-dependent duality.

Although current therapeutic approaches may alleviate symptoms or slow progression, effective treatments directly targeting underlying fibrogenic mechanisms remain unavailable. Given its regulatory influence on hepatic metabolism and inflammation, particularly through the modulation of HSC activation, GDF15 has emerged as a promising candidate for therapeutic intervention. This review aims to provide a comprehensive overview of the regulatory roles of GDF15 in liver fibrosis and to explore its potential as a biomarker and therapeutic target, thereby offering insights for the development of targeted antifibrotic strategies.

## 2. Liver Fibrosis

### 2.1. Molecular Epidemiology of Liver Fibrosis

Epidemiological studies reveal a growing global burden of liver fibrosis, which has become a leading cause of liver-related morbidity [28]. It is estimated that approximately 200 million individuals worldwide are affected by chronic liver diseases, with a considerable subset progressing to advanced fibrosis [29]. A recent systematic review and meta-analysis shows that the pooled prevalence of advanced liver fibrosis in adults aged 18 and above is approximately 3.3% (95% CI: 2.4–4.2%), with substantial variation observed across different geographic regions [30]. The asymptomatic nature of early-stage liver fibrosis delays timely diagnosis and treatment, thereby increasing the risk of progression to life-threatening complications such as cirrhosis and HCC [31]. These epidemiological insights underscore the urgent need to elucidate the principal etiological drivers of hepatic fibrogenesis, which is essential for the development of early diagnostic tools and precision therapeutic strategies.

### 2.2. Etiology and Pathogenesis of Liver Fibrosis

#### 2.2.1. Virus Infections

**Figure 1:**
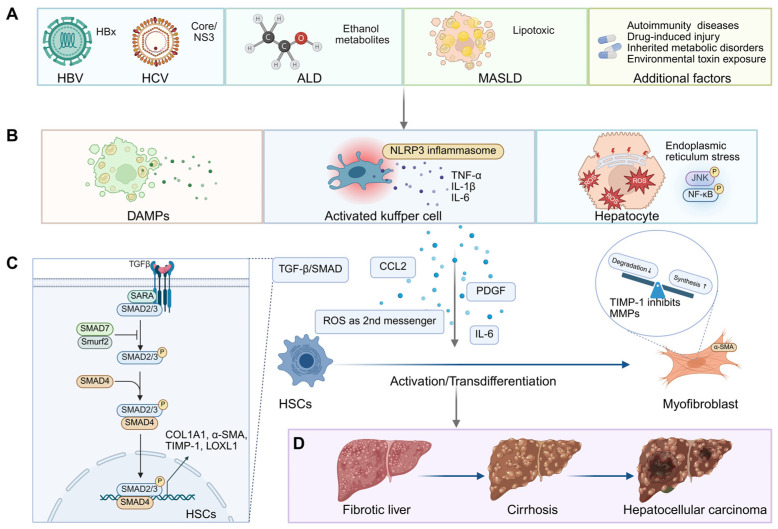
The pathogenic cascade of liver fibrosis: from etiologies to disease progression. (**A**) Diverse etiologies initiate chronic liver injury through specific molecular triggers. Key factors include the following: ① Viral hepatitis: Hepatitis B virus (HBV) and hepatitis C virus (HCV, via Core/NS3 proteins) directly promote stellate cell activation and inflammatory signaling. ② Toxic/metabolic injury: Alcohol-related liver disease (ALD, mediated by ethanol metabolites). ③ Metabolic dysfunction-associated steatotic liver disease (MASLD, driven by lipotoxicity). ④ Other insults: Autoimmune diseases, drug-induced liver injury, inherited metabolic disorders, and environmental toxin exposure. (**B**) Damage-associated molecular patterns (DAMPs) from injured hepatocytes activate Kupffer cells, triggering NOD-like receptor family pyrin domain-containing 3 (NLRP3) inflammasome assembly and secretion of pro-inflammatory cytokines (tumor necrosis factor-alpha [TNF-α], interleukin-1 beta [IL-1β], interleukin-6 [IL-6]). Concurrent endoplasmic reticulum (ER) stress in hepatocytes activates c-Jun N-terminal kinase (JNK) and nuclear factor kappa-light-chain-enhancer of activated B-cell (NF-κB) pathways, exacerbating inflammatory and metabolic injury. (**C**) Core profibrogenic signaling pathways converge on hepatic stellate cell (HSC) activation. Key mediators include: ① Transforming growth factor-beta (TGF-β)/sma- and mad-related proteins (SMAD): Central pathway driving fibrogenesis. ② Chemokine/cytokine signaling: C-C motif chemokine ligand 2 (CCL2) and platelet-derived growth factor (PDGF) promote HSC migration and proliferation. ③ Oxidative stress: Reactive oxygen species (ROS) act as critical second messengers. These signals upregulate expression of fibrogenic markers (collagen type I alpha 1 [COL1A1], alpha-smooth muscle actin [α-SMA], tissue inhibitor of metalloproteinases 1 [TIMP-1], lysyl oxidase-like 1 [LOXL1]). TIMP-1 inhibits matrix metalloproteinases (MMPs), preventing extracellular matrix (ECM) degradation. (**D**) HSC transdifferentiation and disease progression. Activated HSCs undergo phenotypic transformation into collagen-producing myofibroblasts. Sustained activation leads to excessive ECM deposition, resulting in sequential pathological stages: fibrotic liver-liver cirrhosis-hepatocellular carcinoma (HCC).

Chronic viral infections, particularly hepatitis B (HBV) and hepatitis C (HCV), are established as major etiological factors in liver fibrosis [32]. According to the World Health Organization, approximately 254 million people are living with chronic HBV infection, with about 1.2 million new cases reported annually [33]. Similarly, nearly 50 million people are affected by chronic HCV, with roughly 1 million new infections estimated to occur each year [34]. These viruses promote fibrotic progression through the release of damage-associated molecular patterns (DAMPs), which elicit persistent immune activation and stimulate profibrotic signaling pathways (Figure 1) [35,36,37,38]. In HBV infection, the viral X protein is known to activate c-Jun N-terminal kinase (JNK) and ERK signaling and to upregulate special AT-rich sequence-binding protein 1 (SATB1) expression, thereby facilitating the paracrine activation of HSC. SATB1 further drives fibrogenesis by inducing the production of profibrotic mediators such as interleukin (IL)-6, connective tissue growth factor, and platelet-derived growth factor-A [39]. In HCV infection, core and NS3 proteins induce calcium influx and reactive oxygen species (ROS) production in HSC; the core protein additionally upregulates intercellular adhesion molecule-1 expression [40]. Notably, HCV can directly infect hepatic myofibroblasts, triggering their profibrotic activation, which is characterized by differentiation, proliferation, and excessive collagen production, a process that directly contributes to ECM accumulation [41].

#### 2.2.2. Alcohol-Related Liver Disease

Alcohol-related liver disease (ALD) is recognized as another leading cause of liver fibrosis worldwide [42]. Chronic alcohol consumption drives fibrogenesis through multiple interconnected mechanisms [43]. First, alcohol metabolism generates excess ROS, resulting in oxidative stress and hepatocellular injury [44]. Second, signals released from injured and apoptotic hepatocytes stimulate ECM deposition, accelerating fibrotic remodeling [45,46]. Third, alcohol-induced hepatic inflammation activates resident immune cells such as Kupffer cells and HSC, which secrete pro-inflammatory cytokines that promote HSC activation and their transformation into collagen-producing myofibroblasts [47]. Emerging evidence indicates that binge alcohol intake accelerates metabolic dysfunction-associated steatohepatitis (MASH) fibrosis via neutrophil extracellular traps, which activate NOD-like receptor family, pyrin domain-containing 3 (NLRP3)-dependent profibrotic pathways in HSC and monocytes [48]. Additionally, alcohol-induced epigenetic dysregulation imprints a persistent profibrotic memory in the liver, contributing to an irreversible fibrotic phenotype even after the cessation of alcohol consumption, a hallmark of ALD pathogenesis [49].

#### 2.2.3. Metabolic Dysfunction-Associated Steatotic Liver Disease

Metabolic dysfunction-associated steatotic liver disease (MASLD, formerly known as NAFLD), a metabolic liver disease associated with obesity, insulin resistance, and dyslipidemia, affects nearly one-quarter of the global population [50,51]. Fibrogenesis in MASLD is initiated by lipotoxic hepatocyte injury, DAMP-mediated NLRP3 inflammasome activation in Kupffer cells, and subsequent TGF-β/PDGF-driven trans-differentiation of HSC (Figure 1). This process is further exacerbated by impaired autophagy and epigenetic suppression of resolution pathways [52]. Endoplasmic reticulum (ER) stress plays a critical role by activating JNK and nuclear factor kappa-light-chain-enhancer of activated B-cell (NF-κB) pathways, thereby promoting metabolic inflammation and hepatic insulin resistance [53]. In this insulin-resistant environment, the activation of sterol regulatory element-binding protein 1c (SREBP-1c) and sterol regulatory element-binding protein 2 (SREBP-2) enhances de novo lipogenesis and very low-density lipoprotein (VLDL) assembly. The resulting lipid accumulation further aggravates ER stress and lipotoxicity, creating a self-perpetuating pathogenic cycle that ultimately induces hepatocyte apoptosis [53,54]. Approximately 20–30% of MASLD patients progress to MASH, a more aggressive inflammatory phenotype that significantly increases the risk of progressive fibrosis, cirrhosis, and end-stage liver complications [55]. In MASH, lipotoxic metabolites and oxidative stress increase hepatocyte vulnerability, leading to cell-cycle arrest and upregulation of apoptotic markers such as caspases and Fas receptors. Ballooned hepatocytes evade apoptosis through Hedgehog signaling-mediated caspase inhibition while simultaneously secreting pro-inflammatory and profibrogenic mediators. This maladaptive survival response culminates in necrotic cell death, amplifying tissue injury and accelerating fibrotic progression [56].

#### 2.2.4. Additional Etiological Factors

Other contributors to liver fibrosis include autoimmune liver diseases, drug-induced liver injury, inherited metabolic disorders, and environmental toxin exposure [57,58,59]. Additionally, chronic systemic conditions such as cardiovascular disease, hypertension, and diabetes are increasingly recognized as comorbidities that exacerbate hepatic fibrogenesis [50,60,61]. Current therapeutic strategies focus on targeting diverse pathways involved in HSC activation, metabolic reprogramming, and epigenetic regulation, often through combination approaches. However, due to the complex and multifactorial nature of fibrogenesis, most interventions remain in preclinical stage, and no therapy has yet demonstrated consistent and significant antifibrotic efficacy in clinical settings [62]. Therefore, early identification of individuals at high risk of fibrotic progression and the implementation of timely, mechanism-based interventions are crucial for effective management and mitigation of liver fibrosis.

### 2.3. Molecular Mechanisms of Liver Fibrosis

#### 2.3.1. TGF-β: The Central Signaling Axis in Liver Fibrosis Progression

Research has established that the pathogenesis and progression of liver fibrosis involve a complex interplay of multiple pathological mechanisms, including hepatocellular injury, dysregulated immune responses, persistent activation of HSC, disruption of ECM homeostasis, and intestinal microbiota dysbiosis [63]. The core pathophysiological hallmark is the aberrant activation of HSC, driven by phenotypic transformation and an imbalance between ECM synthesis and degradation [64]. In the healthy liver, HSC reside in the perisinusoidal space and function primarily in storing vitamin A and its metabolites [65]. However, in response to liver injury or inflammatory stimuli (such as chronic viral infection, alcoholic liver disease, or MASLD), damaged hepatocytes release DAMPs, which activate HSC through signaling pathways including TGF-β/sma- and mad-related proteins (SMAD), wingless-related integration site (Wnt)/β-catenin, and janus kinase (JAK) signal transducer and activator of transcription (STAT) [66]. Upon activation, HSC undergo marked morphological changes, including elongation and flattening, and begin to express α-smooth muscle actin (α-SMA) [67]. They also initiate synthesis and secretion of ECM components, particularly type I collagen (Col-I) and type III collagen (Col-III) [68]. Sustained activation drives HSC trans-differentiation into myofibroblast-like cells, resulting in excessive ECM deposition and liver fibrosis [65].

Notably, SMAD3-dependent signaling activated by transforming growth factor-β1 (TGF-β1) represents a central molecular mechanism in liver fibrosis pathogenesis [69]. TGF-β regulates cell growth, immune responses, and tissue repair [70]. The TGF-β superfamily consists of TGF-β1, TGF-β2, and TGF-β3, all of which exhibit profibrotic activity, with TGF-β1 being the most potent and well-characterized inducer of fibrosis [71]. TGF-β1 is primarily produced by hepatocytes and Kupffer cells [72]. During liver fibrosis, it activates HSC and promotes ECM accumulation, serving as the principal stimulus for collagen-producing cells in chronic liver injury [72,73,74]. Specifically, TGF-β1 binds to TGF-β receptor II (TβRII), which then phosphorylates and activates TGF-β receptor I (TβRI). This leads to phosphorylation of SMAD3, a key mediator of fibrotic responses. Phosphorylated SMAD3 upregulates the expression of matrix metalloproteinase-1 (MMP1), α-SMA, and Col-I, while also promotes lysyl oxidase-like 1 (LOXL1) overexpression, collectively accelerating fibrosis progression [75]. Moreover, TGF-β1-induced SMAD3 activation enhances tissue inhibitor of metalloproteinase-1 (TIMP-1) expression, which inhibits ECM degradation and further promotes fibrogenesis [76,77]. Importantly, it has been demonstrated that extracellular matrix protein 1 (ECM1) exerts hepatoprotective effects by inhibiting latent TGF-β1 activation, suggesting its potential as a novel therapeutic agent for liver fibrosis [78].

#### 2.3.2. Inflammatory Mechanisms in Liver Fibrosis

Persistent hepatic injury drives fibrogenesis largely through dysregulated inflammatory signaling and cytokine networks, establishing a self-perpetuating cycle. Chronic insults such as viral infections, ALD, and MASLD induce ongoing hepatocyte damage and death [63]. This ongoing injury leads to the release of DAMPs and, in the context of infection, pathogen-associated molecular patterns (PAMPs) [79,80]. These factors activate pathogen recognition receptors, including membrane bound Toll-like receptors and intracellular nucleotide binding oligomerization domain-like receptors, which are expressed on various immune cells as well as parenchymal cells in the liver [81,82]. DAMPs and PAMPs potently activate resident Kupffer cells, which together with other immune cells and damaged hepatocytes release proinflammatory cytokines such as tumor necrosis factor-alpha (TNF-α), IL-1β, and IL-6, along with chemokines including C-C motif chemokine ligand 2 (CCL2), C-X-C motif chemokine ligand 1 (CXCL1), and CXCL8 [83,84,85]. These inflammatory mediators further exacerbate hepatocyte injury and apoptosis, amplify the initial damage signals, and recruit and activate additional inflammatory cells including neutrophils, macrophages, and lymphocytes. Furthermore, they strongly stimulate hepatic stellate cell activation, proliferation, and extracellular matrix production [86]. Thus, dysregulated inflammation and sustained cytokine release serve as central drivers in both the initiation and progression of liver fibrosis.

#### 2.3.3. Oxidative and ER Stress in Liver Fibrosis

In addition to inflammation, chronic hepatic injury also induces significant intracellular stress responses, particularly oxidative stress and ER stress, which markedly exacerbate hepatocellular injury and directly promote fibrogenesis [87,88]. Multiple pathogenic factors, including hepatotoxins such as ethanol metabolites and carbon tetrachloride, lipid overload in MASLD or MASH, viral infections, and bile acid accumulation in cholestatic conditions, collectively impair mitochondrial function and deplete endogenous antioxidants like glutathione and superoxide dismutase. Together with inflammatory cytokines such as TNF-α and IL-1β, these stressors disrupt ER homeostasis, resulting in the accumulation of misfolded proteins and triggering the unfolded protein response (UPR) [89]. Both mitochondrial dysfunction and sustained activation of the UPR serve as major sources of excessive ROS production [90]. ROS directly damage essential hepatocyte components, causing lipid peroxidation and oxidative modifications to proteins and DNA, thereby promoting apoptotic cell death and perpetuating DAMP release. This cycle further amplifies the inflammatory response. Significantly, ROS also act as key signaling molecules that activate and upregulate critical fibrogenic markers such as Col-I and α-SMA in these activated cells [63,91,92]. Thus, oxidative and ER stress mechanisms not only cause direct hepatocyte injury but also initiate profibrogenic signaling pathways in hepatic stellate cells, collectively driving the progression of liver fibrogenesis.

## 3. The Biology and Pathological Role of GDF15 in Liver Fibrosis

### 3.1. The Biology of GDF15: Expression and Protein Synthesis

**Figure 2:**
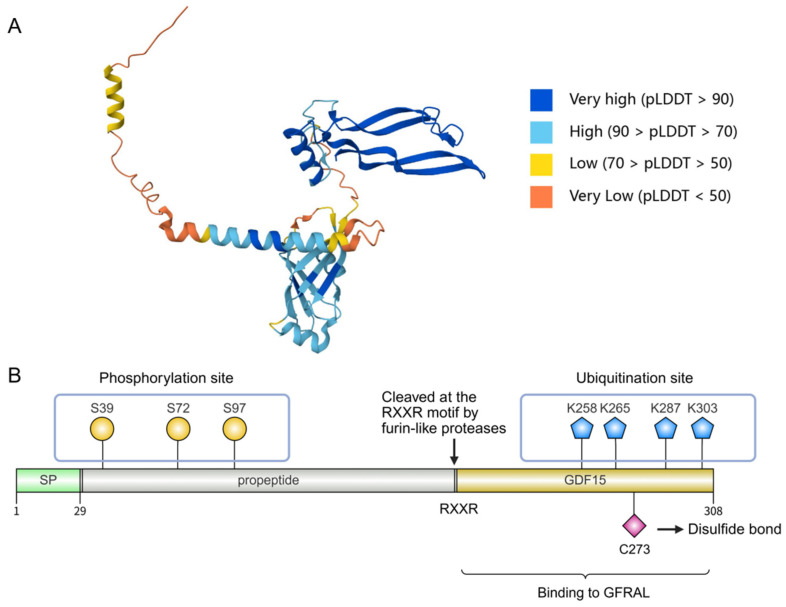
Schematic representation of GDF15 protein structure and its key post-translational modifications. (**A**) Predicted domain architecture of human GDF15 based on AlphaFold (https://alphafold.com/). This model provides a hypothetical structural framework and is not an experimentally determined structure. (**B**) Mapping of functional sites and post-translational modifications. Experimentally confirmed phosphorylation sites (S39, S72, S97) are depicted as solid circles in the propeptide region. Predicted ubiquitination sites (K258, K265, K287, K303) in the mature domain are shown as open circles; among these, K287 and K303 are annotated as critical for stability based on sequence analysis. The predicted disulfide bond at C273 (open diamond) is essential for dimer stabilization. The mature GDF15 dimer binds to its receptor GFRAL to initiate downstream signaling. Note: All sites without explicit experimental references are predictions based on bioinformatic analysis and require further experimental validation.

GDF15 was first identified in 1997 by Bootcov et al., who cloned it from a cDNA library of human monocytic U937 cells. It was characterized as a novel member of the TGF-β superfamily with important functions in macrophage differentiation, activation, and inflammatory responses [11]. Shortly thereafter, Böttner et al. isolated the same molecule from a human placental cDNA library and confirmed its broad expression across various tissues, particularly in epithelial cells and macrophages [93]. Subsequent studies have revealed that GDF15 expression is influenced by diverse factors such as tissue type, injury, regeneration, and inflammation. The protein structure of GDF15 is depicted in Figure 2A (from AlphaFold). Although GDF15 is highly expressed in the adult liver under basal conditions, this expression pattern alone does not establish a direct physiological role in hepatic homeostasis; rather, it may primarily reflect the liver’s role as a source of circulating ligand that acts centrally via GFRAL or as a marker of tissue stress. In fact, GDF15 knockout mice exhibit no obvious defects in liver injury or regeneration [94]. Furthermore, the activity and stability of GDF15 are tightly regulated by post-translational modifications (PTMs), among which phosphorylation and ubiquitination play particularly critical roles. Large-scale phosphoproteomic studies have experimentally identified several serine residues within the GDF15 propeptide region, including S39, S72, and S97, as bona fide phosphorylation sites [95] (Figure 2B). While their presence is established, the functional significance of these modifications requires further experimental validation. Importantly, a central aspect of GDF15 function is its capacity to modulate the phosphorylation status of key downstream signaling proteins. For example, in cervical cancer, GDF15 overexpression or exogenous treatment markedly increases the levels of phosphorylated AKT (p-AKT1) and phosphorylated ERK1/2 (p-ERK1/2), thereby activating the PI3K/AKT and MAPK/ERK pathways to promote cell proliferation [96]. Similarly, in metabolic regulation, recombinant GDF15 enhances hepatic AMPK phosphorylation at Thr172, suppressing gluconeogenesis and ameliorating metabolic dysfunction [97]. In addition, based on sequence analysis and consensus motif searches, several putative ubiquitination sites (K258, K265, K287, K303) have been mapped to the mature domain, with K287 and K303 predicted to be critical for regulating protein stability (Figure 2B). These predictions, however, await experimental verification. Collectively, these findings indicate that GDF15 primarily functions as an extracellular cue that initiates distinct phosphorylation cascades in target cells, rather than relying on its own phosphorylation for bioactivity.

**Figure 3:**
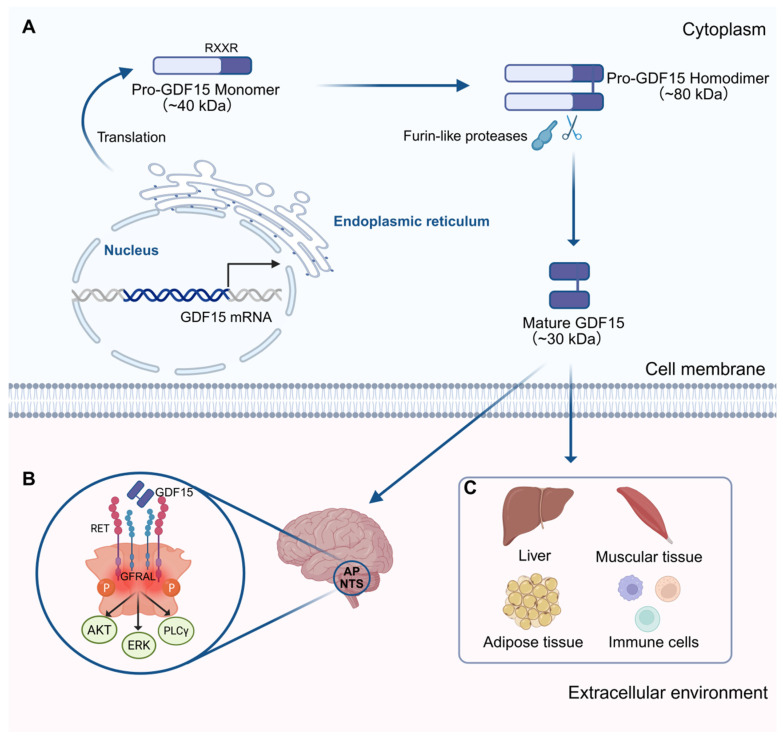
Biosynthesis, receptor interaction, and systemic functions of growth differentiation factor 15 (GDF15). (**A**) GDF15 synthesis and secretion. The mature, secreted form of GDF15 is a ~30 kDa disulfide-linked homodimer. It is produced from a ~40 kDa precursor through a multi-step process involving dimerization, and furin-mediated cleavage of the pro-domain. (**B**) High-affinity binding to the central GFRAL-RET receptor complex. The biological actions of GDF15 are mediated by its high-affinity receptor GDNF family receptor alpha-like (GFRAL), exclusively expressed in brainstem neurons. Upon binding to GFRAL, the co-receptor rearranged during transfection (RET) is activated, thereby triggering intracellular phosphorylation cascades such as protein kinase B (AKT), extracellular signal-regulated kinase (ERK1/2) and phospholipase C gamma (PLCγ). (**C**) Diverse effects of GDF15 in peripheral tissues. GDF15 mediates a broad spectrum of functions in multiple peripheral tissues, such as the liver, skeletal muscle, adipose tissue and immune cells.

As a divergent member of the TGF-β superfamily, GDF15 exhibits distinct synthesis and activation mechanisms (Figure 3A). Similar to other TGF-β proteins, GDF15 is initially synthesized within the ER as an inactive precursor, a pro-GDF15 monomer of approximately 40 kDa. This precursor consists of 167 amino acids and contains an N-linked glycosylation site at residue [76,98]. The monomer forms a homodimer (~80 kDa) through disulfide bonding and is proteolytically cleaved at the RXXR motif by furin-like proteases. This cleavage yields a mature C-terminal dimer (~30 kDa) [99,100]. The mature dimer is regarded as the biologically active form of GDF15.

GDF15 stability and secretion are critically regulated by ubiquitination, a key post-translational modification. Studies have mapped specific lysine residues critical for GDF15 ubiquitination, including K258, K265, K287, and K303, within the mature domain [101,102]. Among these, K287 and K303 appear to be particularly important, as mutation of these residues markedly attenuates GDF15 ubiquitination and degradation [103]. Hepatic glucuronyl C5-epimerase (Glce) has been identified as a key stabilizer of GDF15. Glce interacts directly with GDF15 and suppresses its K48- and K63-linked polyubiquitination, thereby preventing proteasomal and lysosomal degradation and stabilizing mature GDF15 levels [103]. The steady-state level of the GDF15 protein reflects a balance between its synthesis and degradation, and this balance can be disrupted under pathological conditions. For instance, under high-glucose stress, GDF15 protein expression is markedly suppressed in human dermal fibroblasts, a decrease correlated with transcriptional downregulation [104].

### 3.2. GFRAL: The High-Affinity Receptor for GDF15 and Its Diverse Biological Functions

In 2017, GDF15 was identified as the high-affinity receptor for GFRAL by several independent research groups [19,20,105]. GFRAL, encoded by the GFRAL gene located on the short arm of chromosome 6, consists of 394 amino acid residues and has a molecular mass of 44.5 kDa. It is a membrane-anchored protein featuring an extracellular N-terminal domain and a single transmembrane helix that anchors the C-terminus intracellularly [23]. Key residues within GFRAL, including Leu132, Ala135, Glu136, Val139, Gly140, Val142, Asn145, Ala149, Leu152, Lys153, Ile196, Pro197, Gln200, Ser201, and Ala204, are critical for GDF15 binding. Among these, Glu136, Asn145, and Gln200 play essential roles in forming hydrogen bonds with GDF15 [18].

The prevailing view suggests that GFRAL serves as the main binding receptor for GDF15 in the central nervous system, primarily acting through its interaction with the RET tyrosine kinase co-receptor [105] (Figure 3B). Expression of GFRAL is largely restricted to neurons within the area postrema (AP) and the nucleus tractus solitarius (NTS) in the brainstem [106]. Mechanistic studies have shown that the binding of GDF15 to the GFRAL–RET complex induces RET phosphorylation, leading to activation of downstream pathways such as ERK1/2, AKT, phospholipase C gamma (PLCγ), and c-Fos [19,107]. In situ hybridization has confirmed the co-expression of GFRAL and RET in these brain regions [107].

While the canonical GDF15-GFRAL-RET axis plays an essential role in mediating central anorectic and metabolic responses, accumulating evidence indicates that GDF15 also exerts direct peripheral effects. Studies in knockout mice have shown that GDF15 regulates lipid metabolism in subcutaneous adipose tissue independently of GFRAL. Specifically, GDF15 deficiency, but not GFRAL deficiency, alters the expression of key lipogenic enzymes in this depot. Whether GDF15 exerts comparable peripheral effects in other tissues, such as the liver, skeletal muscle, and immune cells, remains an open question [108,109,110,111,112] (Figure 3C). As an illustration, in vitro studies in cardiomyocytes have suggested that GDF15 may signal through TGF-β receptor 2 and SMAD pathways, raising the possibility of additional GFRAL-independent mechanisms in the cardiovascular system [113,114]. In the liver, GDF15 activates AMPK and suppresses gluconeogenesis in both mouse models and primary hepatocytes. These findings raise the possibility that GDF15 directly regulates hepatic metabolism independently of its central GFRAL-mediated actions [97]. Notably, GDF15 impairs T-cell adhesion and extravasation by interfering with LFA-1/ICAM-1 interactions independently of GFRAL, a mechanism supported by in vitro adhesion assays and validated in syngeneic and humanized mouse tumor models, thereby limiting T-cell infiltration and contributing to resistance against PD-1-based immunotherapy [115]. This immunosuppressive function has been clinically validated in patients with anti-PD-1/PD-L1 refractory cancers, where GDF15 neutralization restored antitumor immune responses [116]. In neutrophils, GDF15 inhibits chemokine-induced β2 integrin activation, adhesion, and transendothelial migration, and this effect has been demonstrated by in vitro adhesion assays under flow conditions and confirmed in vivo using a murine myocardial infarction model, in which GDF15 deficiency led to excessive neutrophil recruitment and increased cardiac rupture [117]. Notably, this effect is mediated by the transforming growth factor-β receptor I/II (TGF-βRI/II) heterodimer, as supported by in vitro pharmacological inhibition using small-molecule inhibitors and neutralizing antibodies, siRNA-mediated knockdown, and in vivo studies in neutrophil-specific conditional knockout mice for either Alk5 or Tgfbr2, which recapitulated the enhanced neutrophil extravasation phenotype observed in GDF15-deficient animals [118]. Together, these findings support a conceptual framework in which GDF15 signaling can be broadly categorized into GFRAL-dependent central effects and GFRAL-independent peripheral mechanisms, with the latter being firmly supported by evidence in adipose tissue and immune cells, with emerging evidence in the liver suggesting a similar possibility.

### 3.3. GDF15 as a Biomarker and Protective Mediator in Liver Fibrosis

Under physiological conditions, GDF15 expression remains low; however, it is markedly upregulated in response to liver injury and inflammation [15]. Accumulating evidence supports its role as a clinically relevant biomarker across various liver diseases. Notably, GDF15 has been identified as a potential serum biomarker for HBV-related liver pathology and is also elevated in patients with chronic HCV infection, indicating its involvement in viral hepatitis-induced liver injury [119,120,121]. Furthermore, clinical studies have reported a positive correlation between serum GDF15 levels and the severity of MASLD, particularly in cases with advanced fibrosis [122]. Significantly elevated GDF15 levels are also observed in patients with MASH and autoimmune hepatitis (AIH) [123].

Beyond these disease-specific observations, recent longitudinal and multiomics studies have substantially strengthened the clinical evidence supporting GDF15 as a noninvasive biomarker for liver fibrosis. A recent six-year retrospective cohort study further extended previous cross-sectional observations by demonstrating that elevated baseline circulating GDF15 independently predicted subsequent progression of hepatic steatosis and liver fibrosis. These findings suggest that GDF15 is not merely a marker reflecting existing liver injury but may also serve as a dynamic indicator of disease progression, supporting its potential utility for longitudinal monitoring and risk stratification in patients with chronic liver diseases [124]. More recently, an integrated translational study combining proteomics, transcriptomics, single-cell RNA sequencing, experimental validation in mouse models, and clinical cohort analyses consistently identified GDF15 as a high-confidence circulating biomarker for liver fibrosis. Importantly, GDF15 demonstrated excellent diagnostic performance (AUC > 0.94), outperforming conventional noninvasive indices such as FIB-4. Moreover, incorporation of GDF15 into a multivariable prediction model further improved fibrosis stratification, highlighting its potential application in precision diagnosis and individualized risk assessment [125]. Beyond fibrosis staging, recent clinical evidence suggests that GDF15 also possesses prognostic value in advanced chronic liver disease. In a prospective cohort of patients with cirrhosis, elevated circulating GDF15 was independently associated with liver stiffness, portal hypertension, bacterial translocation, hepatic decompensation, and both liver-related and all-cause mortality, indicating that GDF15 may serve as an integrated biomarker reflecting disease activity and clinical prognosis [126]. Collectively, these recent longitudinal and multiomics studies substantially strengthen the evidence supporting the role of GDF15 as a clinically relevant biomarker. Beyond reflecting fibrosis severity, GDF15 appears capable of predicting disease progression and improving noninvasive risk stratification, thereby reinforcing its translational potential for precision management of chronic liver diseases.

In addition to its diagnostic utility, accumulating experimental evidence suggests that GDF15 also participates directly in the regulation of fibrogenesis. In the early stages of liver fibrosis, GDF15 upregulation may represent a compensatory mechanism aimed at mitigating tissue damage. However, as fibrosis advances, GDF15 levels continue to rise and exhibit a positive correlation with the degree of fibrosis [127]. The highest expression levels are observed in cirrhosis and HCC [27,128]. Mechanistic insights into the protective role of GDF15 have been provided by animal studies. Jurado-Aguilar et al. demonstrated that GDF15−/− mice display elevated hepatic TGF-β1 levels and enhanced SMAD3 phosphorylation, accompanied by exacerbated fibrotic phenotypes. Treatment with recombinant GDF15 (rGDF15) or the SMAD3 inhibitor SIS3 was found to significantly attenuate fibrosis progression, suggesting that GDF15 exerts antifibrotic effects by inhibiting the TGF-β1/SMAD3 pathway [97]. Additionally, Gong et al. reported that GDF15 activates the Nrf2-antioxidant response element (Nrf2-ARE) pathway via suppression of TGF-β/SMAD signaling. This enhances the expression of antioxidant enzymes, improves cellular redox capacity, and reduces oxidative stress and inflammatory responses, thereby further protecting against hepatocyte injury and fibrosis development [129]. Beyond its established role as a secreted cytokine, emerging evidence indicates that the intracellular precursor form of GDF15 (pro-GDF15) exerts distinct antifibrotic functions through receptor-independent mechanisms. Pro-GDF15 contains functional nuclear localization and export signals, allowing it to translocation into the nucleus, where it directly interacts with components of the SMAD transcriptional complex. Nuclear pro-GDF15 has been shown to disrupt the binding of SMAD complexes to their DNA target elements, without affecting SMAD phosphorylation or nuclear translocation. This interaction attenuates TGF-β1-induced transcriptional activity, leading to reduced expression of profibrotic genes and alterations in cell migration and invasion [130]. Recent studies further indicate that nuclear pro-GDF15 accumulation is required for the subsequent secretion of mature GDF15, and that blocking its nuclear export can inhibit mature GDF15 release, suggesting a critical link between nuclear translocation and extracellular activity [99]. These results reveal an additional layer of GDF15 signaling in which pro-GDF15 functions as a nuclear modulator of TGF-β/SMAD-dependent transcription.

Recent reviews further emphasize that GDF15 exerts context-dependent and compartment-specific biological effects. In particular, nuclear pro-GDF15 acts as an endogenous inhibitor of TGF-β1 signaling, a role that is distinct from the well-characterized endocrine and paracrine actions of mature GDF15 [131]. Together, these results suggest that GDF15 may alleviate liver fibrosis through multiple complementary mechanisms, including the suppression of extracellular TGF-β/SMAD signaling and direct nuclear interference with profibrotic transcriptional programs. This dual functionality underscores the value of GDF15 not only as a biomarker of disease progression but also as a multifaceted biological mediator in the pathogenesis and potential treatment of liver fibrosis. The detailed mechanisms underlying these antifibrotic effects are further discussed in the following section.

## 4. GDF15 in Liver Fibrosis: Cell-Specific Effects

In recent years, GDF15 has garnered increasing attention for its regulatory roles in key processes underlying liver fibrosis progression, including HSC activation and the function of immune cells. HSC, as the primary source of myofibroblasts, play a central role in the development of liver fibrosis. Furthermore, immune cells, such as T cells, macrophages, natural killer (NK) cells and B cells, significantly contribute to both the initiation and resolution of fibrotic processes. Additionally, mesenchymal stem cells (MSCs) demonstrate antifibrotic potential via immunomodulatory effects and paracrine signaling mechanisms (Table 1). Thus, by modulating the hepatic microenvironment, GDF15 represents a promising therapeutic target for liver fibrosis.

### 4.1. GDF15 Inhibits Liver Fibrosis by Suppressing the Activation of HSC

**Figure 4:**
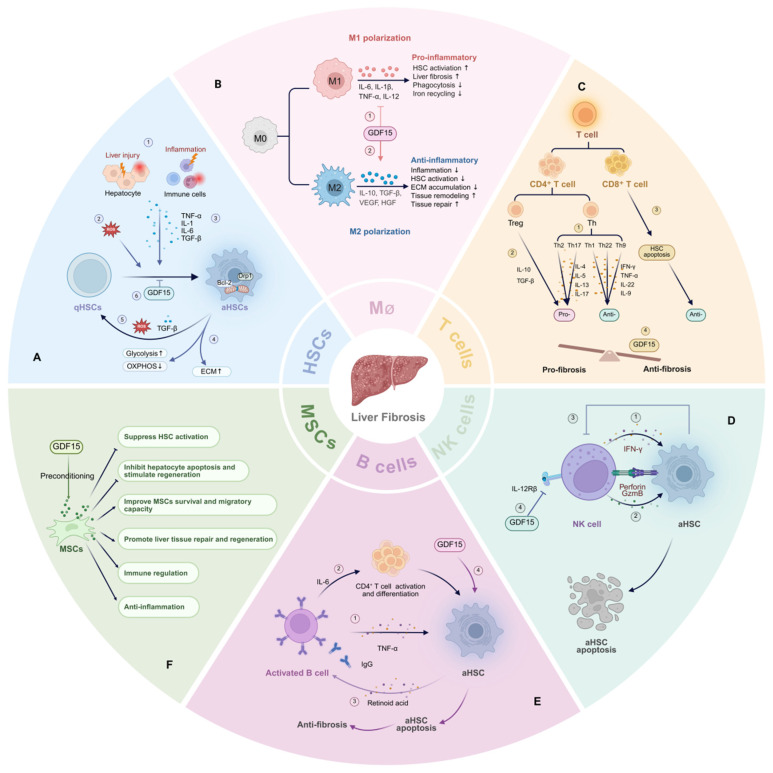
The regulatory role of growth differentiation factor 15 (GDF15) in the liver fibrosis microenvironment. (**A**) Inhibition of hepatic stellate cell (HSC) activation. This schematic illustrates the central mechanisms underlying HSC activation and the progression of liver fibrosis, highlighting the inhibitory role of GDF15. ① Hepatic injury triggered by various etiologies leads to hepatocyte damage and the recruitment of immune cells. These cells release pro-inflammatory cytokines (tumor necrosis factor-alpha [TNF-α], interleukin-1 [IL-1], interleukin-6 [IL-6]) and transforming growth factor-beta (TGF-β), which directly activate quiescent HSC (qHSC). ② Concurrently, elevated reactive oxygen species (ROS) from damaged cells further promote the activation of HSC (aHSC). ③ Upon activation, HSC undergo key intracellular changes including an anti-apoptotic shift via Bcl-2 upregulation, which suppresses mitochondria-mediated apoptosis and promotes survival; Concurrently, increased dynamin-related protein 1 (Drp1)-mediated mitochondrial fission leads to mitochondrial fragmentation and electron transport chain dysfunction. ④ The trans-differentiation of aHSC into myofibroblasts confers a highly proliferative phenotype. This activated state is characterized by a metabolic shift from oxidative phosphorylation (OXPHOS) to glycolysis, and by excessive Col1α1 deposition via TGF-β/SMAD signaling that drives extracellular matrix (ECM) accumulation and fibrosis. ⑤ A critical feed-forward loop, driven by ROS from dysfunctional mitochondria and TGF-β from activated HSC and other cells, perpetuates HSC activation and fibrosis. ⑥ GDF15 acts as a critical antifibrotic modulator by attenuating mitochondrial ROS production, enhancing PTEN-induced kinase 1 (PINK1)-Parkin-mediated mitophagy, promoting an OXPHOS-dominant metabolic phenotype in macrophages, and downregulating key fibrogenic genes. (**B**) Reprogramming of macrophage phenotype and function. ① GDF15 inhibits the M1-like macrophage phenotype, reducing pro-inflammatory cytokine release. ② GDF15 promotes the M2-like phenotype, characterized by secretion of interleukin-10 (IL-10), TGF-β, vascular endothelial growth factor (VEGF), and hepatocyte growth factor (HGF). (**C**) Regulation of T-cell responses. Profibrotic CD4^+^ T helper subsets (Th2, Th9, Th17) directly activate HSC and stimulate ECM deposition. Antifibrotic subsets such as Th1 and Th22 cells antagonize this process. Regulatory T cells (Treg) promote fibrosis through IL-10 and TGF-β. CD8^+^ tissue-resident memory T cells (CD8^+^ Trm) contribute to fibrosis resolution by triggering HSC apoptosis. GDF15 suppresses profibrotic T-cell functions while potentiating Treg immunosuppression. (**D**) Interaction with natural killer (NK) cells. During early injury, NK cells secrete interferon-gamma (IFN-γ) to promote apoptosis of activated HSC and suppress TGF-β signaling, while also directly eliminating HSC via perforin/granzyme B-mediated cytotoxicity. GDF15 suppresses NK cell function through TGF-β receptor I (TGF-βRI)/SMAD1/5/8 pathway, downregulating interleukin-12 receptor beta (IL-12Rβ) and impairing IFN-γ production. (**E**) Synergistic crosstalk with B cells. Activated B cells promote HSC activation via IL-6 and TNF-α. Chronic injury triggers B-cell activation and autoantibody production. Activated HSC secrete retinoic acid to further promote B-cell activation, forming a self-perpetuating cycle. GDF15-mediated apoptosis of aHSC disrupts this feedback loop. (**F**) Enhancement of mesenchymal stem cell (MSC) therapy. GDF15 preconditioning improves MSC survival, migration, and paracrine function.

The hepatic inflammatory microenvironment is critically implicated in the activation of HSC (Figure 4A). Pro-inflammatory cytokines, such as TNF-α, IL-1, and IL-6, released by damaged hepatocytes and immune cells, not only directly promote HSC activation but also exacerbate liver fibrosis by enhancing oxidative stress [91,132]. Elevated levels of ROS impair cellular structures and disrupt metabolic homeostasis via mitogen-activated protein kinase (MAPK) and NF-κB signaling pathways, further driving HSC activation [133,134,135]. Additionally, several other pathways are implicated in regulating HSC autophagy, including the adenosine monophosphate-activated protein kinase (AMPK)/mechanistic target of the rapamycin pathway (mTOR) pathway, ER stress-induced unfolded protein response (UPR), advanced glycation end-products (AGEs), hypoxia-inducible factor-1α (HIF-1α), and extracellular ATP [63]. Moderately enhanced autophagy facilitates the degradation of lipid droplets and release of fatty acids to meet energy demands, while simultaneously promoting the trans-differentiation of HSC into myofibroblasts [136,137]. During fibrogenesis, HSC often upregulate the anti-apoptotic protein Bcl-2 and suppress mitochondria-mediated apoptosis, thereby inhibiting the caspase cascade and enhancing their survival [138,139]. Increased dynamin-related protein 1 (Drp1) activity also promotes mitochondrial fission and fragmentation in HSC, exacerbating mitochondrial dysfunction and sustaining their activated phenotype, which accelerates liver fibrosis progression [140].

Mitochondrial dysfunction disrupts cellular energy metabolism, leading to a metabolic shift in activated HSC characterized by enhanced glycolysis and reduced oxidative phosphorylation (OXPHOS) [141]. This reprogramming redirects energy production from mitochondrial respiration to glycolysis, thereby supporting HSC activation and proliferation [142]. Moreover, electron leakage from the mitochondrial electron transport chain (ETC) results in excessive ROS generation, which induces lipid peroxidation, protein oxidation, and DNA mutations, further aggravating hepatocellular injury and inflammation [143]. ROS also activate HSC through TGF-β/SMAD and NF-κB signaling pathways, promoting excessive Col-I deposition and accelerating fibrosis [143,144]. Importantly, mitochondrial DAMPs, chiefly mitochondrial DNA, which are released upon hepatocyte injury and insufficiently cleared due to impaired macrophage efferocytosis, directly activate hepatic stellate cells. This leads to collagen deposition and liver fibrosis progression, a mechanism validated in the serum of patients with nonalcoholic steatohepatitis [144].

In dietary MASH models, GDF15 suppresses liver fibrosis, a process associated with the downregulation of key profibrogenic genes, including Tgfb1, Col1a1, Timp1, Acta2, and osteopontin. This direct inhibitory effect on gene expression was confirmed in vitro, but only when HSC were co-stimulated with TGF-βand treated with pharmacological doses of GDF15 [145]. Genetic ablation of GDF15 in knockout models in both CCl4- and DDC-induced models exacerbates liver fibrosis progression, concomitant with enhanced HSC activation as indicated by markedly elevated α-SMA expression in fibrotic livers [146]. Importantly, GDF15 deficiency does not alter the rate of apoptosis of activated HSC (α-SMA^+^) [146]. These findings collectively suggest that GDF15 deficiency promotes fibrogenesis primarily by enhancing HSC activation. This effect is likely mediated by an aggravated inflammatory milieu and the direct profibrotic influence of GDF15-deficient macrophages on HSC, rather than by impaired HSC apoptosis. Furthermore, GDF15 is markedly upregulated in response to mitochondrial damage, supporting its role as a systemic biomarker of cellular stress. However, direct experimental evidence linking this mitochondrial stress response to the pathogenesis of liver fibrosis is still lacking [147,148]. Huang et al. demonstrated that GDF15 attenuates mitochondrial ROS production by inhibiting mitochondrial fission, thereby improving mitochondrial function [149]. Li et al. reported that GDF15 promotes a shift in macrophage metabolism toward an OXPHOS-dependent phenotype, reducing mitochondrial ROS accumulation and exerting anti-inflammatory effects [146]. Additional evidence shows that GDF15 counteracts OXPHOS inhibition in hepatocytes and enhances both basal and maximal respiratory capacity, thereby facilitating mitochondrial oxidative metabolism [150]. Thus, GDF15 may influence hepatocyte energy metabolism by improving mitochondrial function, enhancing cell survival, and maintaining metabolic homeostasis. Mitochondrial damage-induced oxidative stress activates the mitochondrial unfolded protein response (mtUPR), leading to the upregulation of GDF15 expression. As part of the cellular stress response, GDF15 plays a protective role by promoting adaptation to mitochondrial damage and attenuating liver fibrosis [151]. Moreover, mitochondrial dysfunction often triggers localized inflammation, and GDF15 has been shown to alleviate liver inflammation by suppressing the overactivation of immune cells, thereby reducing pro-inflammatory signaling and inhibiting fibrosis progression [15,152]. Similarly, mitophagy serves as an essential mechanism for maintaining mitochondrial homeostasis [153,154]. Impaired autophagic function leads to accumulation of damaged mitochondria, increased ROS production, and release of inflammatory cytokines, which aggravate oxidative stress and inflammation. This, in turn, promotes sustained HSC activation and ECM deposition, exacerbating liver fibrosis [155,156,157]. GDF15 facilitates the clearance of dysfunctional mitochondria by modulating the PTEN-induced kinase 1–Parkin-mediated mitophagy pathway, thereby helping to maintain mitochondrial homeostasis and attenuating liver fibrogenesis [158].

However, some studies report context-dependent profibrotic effects of GDF15. Peng et al. showed that in carbon tetrachloride (CCl_4_)- and thioacetamide (TAA)-induced murine fibrosis models, the neutralization of GDF15 with specific antibodies significantly attenuated TGF-β-mediated HSC activation and reduced liver fibrosis severity [159]. Similarly, Dong et al. reported that under chemotherapy or hypoxic conditions, HCC cells upregulate GDF15 via MAPK pathway activation. Secreted GDF15 activates ERK1/2 and SMAD3 signaling in HSC, enhancing their proliferation and collagen production [160]. Another study revealed a reciprocal crosstalk within the tumor microenvironment between HCC cells and HSC. Mechanistically, HSC secrete substantial amounts of GDF15 via autophagy, exerting a paracrine effect that promotes HCC proliferation and tumor growth [161]. In turn, HCC cells secrete pro-fibrogenic factors such as TGF-β and PDGF, which activate HSC, drive excessive ECM deposition, and promote liver fibrosis progression. Importantly, the inhibition of HSC autophagy reduced GDF15 secretion, attenuated HSC activation, and consequently suppressed both tumor growth and liver fibrosis [161].

In summary, available evidence suggests that GDF15 modulates HSC activation in a context-dependent manner and may play a crucial role in maintaining mitochondrial function and cellular homeostasis. Although it generally attenuates liver fibrosis by improving mitochondrial metabolism, suppressing ROS production, enhancing mitophagy, reducing inflammation, and inhibiting sustained HSC activation, GDF15 may paradoxically promote HSC activation and exacerbate fibrosis under specific conditions such as in the presence of hepatocellular carcinoma cells or hypoxia. Therefore, context-specific targeting of GDF15 represents a promising therapeutic strategy to suppress HSC activation and mitigate the progression of liver fibrosis. Further investigation into its molecular mechanisms will not only elucidate key pathological processes but also provide new insights into its potential as a therapeutic target.

### 4.2. GDF15 Suppresses Liver Fibrosis by Modulating Macrophage Differentiation and Function

Hepatic macrophages, including the resident Kupffer cells (KCs), are central immune sentinels located within the liver sinusoids and play a pivotal role in maintaining hepatic homeostasis and responding to injury [162,163]. In fibrotic livers, these cells accumulate within scar regions and engage in dynamic crosstalk with activated HSC, critically influencing disease progression [164] (Figure 4B). Given their remarkable functional plasticity, these macrophages serve as key targets for anti-fibrotic mediators. Accumulating evidence identifies GDF15 as a potent regulator of this macrophage–HSC axis.

Pro-fibrotic macrophages were historically defined as classically activated M1 cells [165]. They secrete pro-inflammatory cytokines such as TNF-α, IL-1β and IL-6, which activate HSC and exacerbate liver fibrosis [166,167]. More recent studies have identified disease-associated subsets, notably SPP1^+^ and THBS1^+^ macrophages, that expand in fibrotic niches and exert pro-fibrogenic effects by releasing TNF-α, IL-1β, SPP1 and THBS1 [57,168,169]. In contrast, anti-inflammatory macrophages, often designated M2, along with certain Kupffer cell populations and monocyte-derived mature macrophages, restrain fibrosis through the production of IL-10, TGF-β and matrix-degrading enzymes such as MMP12 and MMP13 [166,168,170]. These cells also release growth factors, including TGF-β, vascular endothelial growth factor and hepatocyte growth factor, which support tissue remodeling and repair [171,172]. Although the traditional M1/M2 dichotomy oversimplifies the in vivo complexity of hepatic macrophages, it provides a useful framework for understanding how GDF15 may shift the overall macrophage balance toward pro-resolution phenotypes [164,173].

Single-cell and spatial transcriptomic technologies have further revealed a much broader spectrum of macrophage subpopulations. These newly identified subsets include lipid-associated macrophages, LYVE1^+^FOLR2^+^TIMD4^+^ resident macrophages and liver capsular macrophages [164,173,174]. The precise functions of these populations in fibrosis are under active investigation. Which of these subsets are directly modulated by GDF15 is an important open question that directly relates to the anti-fibrotic mechanisms discussed below.

Several lines of preclinical evidence indicate that GDF15 may contribute to liver fibrosis progression by modifying Kupffer cells and other macrophage populations. In one study, GDF15 reduces liver inflammation and fibrosis by suppressing the release of pro-inflammatory signals such as TNF-α and IL-6 [146]. In the same preclinical study, GDF15 was shown to promote macrophage polarization toward an anti-inflammatory M2 state and enhances their anti-fibrotic activity by improving oxidative metabolism and reducing harmful reactive oxygen species (ROS) [146]. In addition, GDF15, induced by alcohol- or toxin-mediated mitochondrial stress in hepatocytes has been reported to exert protective effects by suppressing hepatic inflammation. This anti-inflammatory action is linked to the inhibition of key signaling pathways such as NF-κB, JNK, and p38, ultimately mitigating the progression of liver fibrosis [150]. Preclinical evidence from a chronic alcohol exposure model suggests that hepatocyte-derived GDF15 sensitizes inflammatory Kupffer cells to catecholamine by upregulating ADRB2 expression, thereby promoting their apoptosis and alleviating alcohol-induced liver injury and fibrosis [175]. Conversely, GDF15 deficiency exacerbates hepatic inflammation and fibrogenesis, as evidenced by elevated pro-inflammatory cytokines, increased infiltration of macrophages and neutrophils, and heightened NF-κB pathway activation [176]. These changes are associated with a shift in macrophage phenotype towards a pro-inflammatory state and increased nuclear translocation of p65 in fibrotic livers [175]. Notably, liver fibrosis in conditions like MASH increases GDF15 production. This supports its role as a stress-related signaling protein and a potential treatment target [176]. Furthermore, GDF15 modulates the release of inflammatory mediators by affecting the health of THP-1 cells, a monocyte cell line capable of differentiating into macrophages with antifibrotic potential [177,178].

In summary, current evidence highlights the pivotal role of GDF15 in liver fibrosis through its regulation of macrophage polarization and function. Given the newly recognized diversity of macrophages subsets, future studies should aim to clarify the precise effects of GDF15 on specific macrophage populations such as SPP1^+^ macrophages and resident Kupffer cells and their functional states within the fibrotic microenvironment [164,169]. Further investigation remains necessary to fully delineate the cell-specific actions of GDF15 in fibrogenesis.

### 4.3. GDF15 Alleviates Liver Fibrosis by Modulating T-Cell Responses

T cells play critical and often opposing roles in the development and immunoregulation of liver fibrosis [179,180] (Figure 4C). Th1 cells secrete IFN-γ and TNF-α, which upregulate matrix metalloproteinases and promote ECM degradation, thereby attenuating fibrosis [181]. Th22 cells exert antifibrotic effects primarily through IL-22, which suppresses HSC activation [182,183]. In contrast, Th2-derived IL-4, IL-5 and IL-13 stimulate fibroblast proliferation and ECM deposition, exacerbating fibrogenesis [184]. Th9 cells promote HSC activation and collagen deposition via IL-9-mediated signaling [140]. The Th17/IL-17A axis drives HSC activation and fibrosis, and targeting this pathway ameliorates hepatic fibrosis [185,186,187]. Tregs exhibit context-dependent functions. Some Treg subsets mediate immunosuppression via IL-10 and TGF-β, yet their depletion can paradoxically attenuate fibrosis [188]. In MASH, a specific Treg population promotes fibrosis by producing amphiregulin, which signals through EGFR on HSC [189]. However, not all Treg populations are profibrotic. For example, the intrahepatic Treg subset described by Aki et al. suppresses the pro-inflammatory responses of Th1 and Th17 cells, thereby reducing the activation of profibrotic pathways and attenuating fibrosis [190]. Additionally, CD8^+^ tissue-resident memory T cells facilitate fibrosis resolution by inducing HSC apoptosis through FasL-Fas signaling [191]. This functional complexity renders the T-cell compartment both a contributor to fibrogenesis and a potential therapeutic target. Consistent with this concept, GDF15 has recently been shown to recalibrate the hepatic T-cell compartment toward an anti-inflammatory and immunosuppressive state, as detailed below.

Emerging preclinical evidence suggests that GDF15 may exert antifibrotic effects by modulating T-cell activation. In one study utilizing a CCl_4_-induced murine model, pro-inflammatory CD4^+^/CD44^+^ and CD8^+^/CD44^+^ T cells were elevated, secreting IFN-γ and TNF-α that recruit macrophages and neutrophils and activate HSC, thereby exacerbating fibrosis. In that same study, GDF15 was found to reduce the abundance of these subsets, attenuating immune-driven HSC activation and ameliorating liver injury [150]. Additionally, GDF15 was reported to increase naïve CD4^+^/CD62L^+^ and CD8^+^/CD62L^+^ T-cell populations, promoting hepatic immune homeostasis [150]. It also reduces CD8^+^ TNF-α^+^ T cells, thereby diminishing TNF-α–mediated HSC activation and collagen deposition. Mechanistically, the same study demonstrated that GDF15 suppresses NF-κB, JNK, and p38 MAPK signaling, leading to reduced T-cell activation and lower release of pro-inflammatory cytokines such as IL-6 and TNF-α [150]. Furthermore, GDF15 has been shown in this model to decrease infiltration of CD11b^+^/Ly6C^+^ monocytes and neutrophils, further dampening T-cell activation and ultimately suppressing α-SMA and COL1A1 expression, HSC activation, and ECM deposition [150]. Notably, GDF15 enhances the function of Treg cells through interaction with the surface receptor CD48, which was the first immune receptor to be identified for this ligand. CD48 signaling activates ERK and promotes T-cell activation, whereas GDF15 binding to CD48 regulates fork head box P3 (FOXP3) post-translational modifications. This enhances the differentiation of induced Treg (iTreg) cells and strengthens the immunosuppressive capacity of natural Treg (nTreg) cells [192,193]. Given that Tregs can produce IL-10 and TGF-β to suppress inflammation, the GDF15-CD48-mediated boost in Treg function may contribute to its documented antifibrotic effect [188,192,193].

The immunomodulatory role of GDF-15 in T-cell biology has been increasingly recognized, yet its interplay with immune checkpoint inhibitors (ICIs) within the context of chronic liver disease remains poorly understood. Emerging evidence from cancer immunology has identified GDF-15 as a novel vascular immune checkpoint that functions upstream of classical immune checkpoints by disrupting the LFA-1/ICAM-1 adhesion axis, thereby preventing effector CD8^+^ T-cell extravasation into tissues rather than directly suppressing T-cell activation. Consequently, GDF-15 promotes immune exclusion and confers resistance to PD-1/PD-L1 blockade [115]. This mechanistic concept has recently been validated in the clinic. In the first-in-human GDFATHER-1/2a trial, neutralization of GDF-15 with visugromab combined with nivolumab induced durable clinical responses in patients with checkpoint inhibitor-refractory non-squamous NSCLC and urothelial carcinoma, accompanied by enhanced intratumoral CD8^+^ T-cell infiltration, proliferation, interferon-γ signaling, and granzyme B expression. In addition, pan-cancer transcriptomic analyses identified GDF-15-high tumors as an immune-excluded subtype characterized by diminished cytotoxic T-cell signatures, providing a rationale for biomarker-guided GDF-15-targeted immunotherapy [116]. Collectively, these findings establish GDF-15 as a mechanistically distinct regulator of immune exclusion that complements classical immune checkpoint pathways. Although direct evidence linking GDF-15 to immune checkpoint responsiveness in liver fibrosis is currently lacking, chronic liver fibrosis shares several hallmarks with the tumor microenvironment, including persistent inflammation, sinusoidal vascular remodeling, extracellular matrix accumulation, impaired lymphocyte trafficking, and progressive T-cell dysfunction. These similarities raise the intriguing possibility that GDF-15 may likewise regulate T-cell recruitment and immune checkpoint efficacy during hepatic fibrogenesis. Elucidating the crosstalk between GDF-15 and immune checkpoint pathways may therefore open new avenues for immunotherapeutic intervention in progressive fibrotic liver diseases.

Through these mechanisms, GDF15 attenuates liver fibrosis by recalibrating T-cell responses, shifting the balance from a pro-inflammatory to an anti-inflammatory and immunosuppressive state. The GDF15–T cell axis thus represents a promising immunomodulatory target for the treatment of chronic liver diseases, warranting further investigation into its therapeutic potential.

### 4.4. GDF15 Modulates NK Cell Function to Ameliorate Liver Fibrosis

The interaction between GDF15 and NK cells represents an emerging area of interest in immunology and liver fibrosis research (Figure 4D). As key components of the hepatic innate immune system, NK cells play a central role in antifibrotic immunity. During the early stages of liver fibrosis, NK cells recognize activated HSC through activating receptors, including natural killer group 2 member D (NKG2D) and natural killer cell p46-related protein (NKp46). They subsequently secrete cytokines, particularly interferon-γ (IFN-γ), to exert antifibrotic effects [194,195]. IFN-γ suppresses HSC activation by upregulating pro-apoptotic genes such as BCL2-associated X protein (BAX), thereby inducing HSC apoptosis, while simultaneously inhibiting the TGF-β signaling pathway to reduce ECM synthesis [196]. Additional studies have shown that increased IL-10 expression in fibrotic mice elevates CCL5 levels within the liver, thereby promoting the recruitment of peripheral NK cells to the fibrotic niche and enhancing NKG2D expression on these NK cells. This upregulation strengthens their ability to recognize and eliminate activated hepatic stellate cells. Moreover, IL-10 augments NK-cell secretion of IFN-γ and CD107a, further reinforcing their immunosurveillance capacity and ultimately suppressing the progression of liver fibrosis [197]. NK cells also inhibit HSC activation through prostaglandin E2 receptor 3 (EP3) signaling. Activation of EP3 enhances the cytotoxicity of CD27^+^CD11b^+^ NK cells against activated HSC through protein kinase C (PKC)/Spic-mediated upregulation of Itga4. Increased Itga4 expression promotes vascular cell adhesion molecule 1 (VCAM1)-dependent adhesion, thereby facilitating NK-cell-mediated cytotoxicity [198]. Additionally, NK cells mediate target cell killing through perforin and granzyme release, which activates caspase-3/7 and triggers apoptotic cascades in activated HSC, thereby attenuating fibrosis [198,199]. However, in advanced liver fibrosis, the chronic inflammatory microenvironment impairs NK cell function, characterized by reduced NKp46 expression and diminished cytotoxic capacity. This enables HSC to evade immune surveillance [200]. Moreover, HSC further suppress NK cell activity through TGF-β signaling, fostering hepatic immune tolerance and facilitating fibrosis progression [201].

In systemic inflammation, GDF15 activates the SMAD1/5/8 signaling pathway via TGF-β receptor I (TGF-βRI). This activation downregulates IL-12Rβ, a key receptor required for NK cell responsiveness to IL-12 and subsequent IFN-γ production [202]. One study has proposed that GDF15-mediated suppression may attenuate a critical endogenous anti-fibrotic mechanism in the liver, potentially creating an environment that favors fibrogenesis. This finding suggests a hypothesis that GDF15 may contribute to fibrosis progression by attenuating the pro-inflammatory and cytotoxic functions of NK cells, positioning it as a potential therapeutic target for restoring NK cell activity. Furthermore, immune evasion by activated HSC also plays a crucial role in fibrosis progression. HSC employ multiple strategies to avoid NK cell surveillance, including the downregulation of activating receptor ligands, upregulation of inhibitory receptors, reduction of MHC-I expression, and secretion of immunosuppressive factors. These adaptations promote NK cell exhaustion and contribute to the persistence of fibrotic microenvironments [203].

While NK cells protect against early liver fibrosis by targeting activated HSC and suppressing inflammation, their antifibrotic function becomes markedly impaired during disease progression. GDF15 appears to facilitate this functional decline through suppression of NK cell activity, highlighting its therapeutic relevance. Further investigation remains essential to clarify the detailed mechanisms of GDF15–NK cell crosstalk and to expand our understanding of NK cell-related immune evasion pathways in liver fibrosis.

### 4.5. GDF15 Synergizes with B Cells to Attenuate Liver Fibrosis

B lymphocytes are now firmly established as active contributors to liver fibrosis across various etiologies, including MASH and cholestatic liver diseases [199,200]. In patients with MASLD, intrahepatic B-cell accumulation is significantly increased and correlates with fibrosis severity [204,205]. Experimental studies provide functional evidence for this profibrotic role. In animal models, B-cell deficiency, as observed in μ-chain mutant mice, or antibody-mediated B-cell depletion attenuates liver fibrosis induced by carbon tetrachloride or a methionine-choline-deficient diet. These interventions consistently reduce collagen deposition, inflammatory cell infiltration, and hepatic stellate cell activation [204,205]. Mechanistically, B cells promote fibrogenesis through multiple pathways. They secrete pro-inflammatory cytokines such as TNF-α and IL-6, which directly promote HSC activation, proliferation, and survival [206]. Furthermore, B cells enhance CD4^+^ T-cell activation, thereby amplifying the inflammatory milieu that drives HSC activation [207,208]. This profibrotic immune response is sustained by a positive feedback loop: activated HSC secrete retinoic acid, which promotes further B-cell activation, creating a self-reinforcing cycle that exacerbates ECM deposition and fibrosis progression. Furthermore, chronic liver injury generates neoantigens such as oxidative stress-derived epitopes. B cells activated by these epitopes produce pathogenic autoantibodies, including anti oxidative stress derived epitope IgG, thereby perpetuating a cycle of ongoing injury and fibrosis [209]. Given this significant pathogenic role, B cells have emerged as a promising therapeutic target.

Recent clinical and experimental evidence suggests a synergistic interaction between GDF15 and B cells in the progression and potential treatment of liver fibrosis (Figure 4E). The antifibrotic intervention by GDF15 operates through induction of apoptosis in activated HSC, leading to their direct elimination. aHSCs produce retinoic acid, which drives B-cell activation and sustains a profibrotic inflammatory environment. Targeted inhibition of this signaling pathway thus offers a potential therapeutic avenue to blunt fibrogenesis [206]. Moreover, GDF15 may facilitate innovative cell-based immunotherapies. Secchiero et al. showed that Nutlin-3, a non-genotoxic MDM2 inhibitor, activates p53 signaling in primary B cells and strongly induces pro-apoptotic genes, most notably GDF15. This supports the concept of reprogramming B cells into sustained GDF15 delivery systems [210]. We hypothesize an experimental therapeutic approach in which autologous B cells are pretreated ex vivo with Nutlin-3 to become GDF15-secreting vehicles, then reinfused into patients with liver fibrosis. These engineered cells are anticipated to migrate to fibrotic areas and locally release GDF15, where it may induce apoptosis of activated HSC, suppress their proliferation, and modulate immune cell activity. This remains a hypothetical concept requiring extensive preclinical validation. Additionally, emerging data from studies on COVID-19-related ARDS suggest that GDF15 helps maintain immune homeostasis and supports tissue tolerance [211]. Whether this observation is relevant to the context of liver fibrosis remains entirely speculative and requires dedicated investigation. Based on these collective insights from diverse contexts, we hypothesize that the therapeutic elevation of GDF15 in liver fibrosis may attenuate excessive inflammation, promote regulatory B-cell phenotypes, reduce autoantibody-mediated injury, and enhance the function of beneficial B cells involved in ECM remodeling or the neutralization of profibrotic factors.

Consequently, targeting the GDF15-B cell axis represents a theoretically promising but currently unproven therapeutic strategy for liver fibrosis. This approach may simultaneously suppress HSC activation and restore immune homeostasis, although these hypotheses await experimental validation in liver-specific models.

### 4.6. GDF15 Enhances the Therapeutic Efficacy of MSCs Against Liver Fibrosis

MSCs represent a promising therapeutic approach for liver fibrosis due to their multipotent differentiation capacity, immunomodulatory properties, and paracrine activities (Figure 4F). MSCs secrete various bioactive factors such as VEGF, HGF, epidermal growth factor (EGF), along with ECM remodeling enzymes like MMPs and exosomes containing antifibrotic microRNAs. Together, these secretions inhibit hepatocyte apoptosis, stimulate regeneration, and suppress HSC activation [212,213,214,215]. Under appropriate conditions, MSCs can differentiate into hepatocyte-like cells or fuse with existing hepatocytes, thereby replacing damaged parenchymal cells and promoting tissue repair and regeneration in the fibrotic liver [216,217]. MSCs also exert potent immunomodulatory effects. They prolong neutrophil survival at sites of injury, thereby enhancing the clearance of apoptotic hepatocytes. In addition, MSCs secrete multiple immunoregulatory mediators, including nitric oxide (NO), prostaglandin E2 (PGE2), indoleamine 2,3-dioxygenase (IDO), IL-6, and human leukocyte antigen G isoform 5 (HLA-G5). These mediators suppress T-cell proliferation and regulate the activity of NK cells, macrophages, regulatory T cells, and myeloid-derived suppressor cells (MDSCs) [218,219]. In particular, MSC-derived IL-10 and PGE2 promote macrophage polarization from the pro-inflammatory M1 toward the anti-inflammatory M2 phenotype. This transition reduces the release of proinflammatory cytokines and supports tissue repair [220,221]. Additionally, MSCs alleviate fibrosis by diminishing intrahepatic B-cell infiltration, activation, and production of inflammatory cytokines including TNF-α and IL-6, thereby limiting B cell-dependent HSC activation [222].

Despite their potential, the therapeutic efficacy of MSCs is often limited by their poor survival and reduced functional durability in the harsh microenvironment of the fibrotic liver. Recent evidence suggests that preconditioning strategies, whether genetic or cytokine based, can significantly improve MSCs performance [217]. Notably, GDF15 has emerged as a key candidate for augmenting MSC-based therapies. According to Huang et al., pretreatment with GDF15 lowers mitochondrial ROS generation in MSCs by suppressing excessive mitochondrial fission. This reduction in oxidative stress decreases apoptotic cell death and strengthens paracrine activity. Furthermore, GDF15 stimulates MSC proliferation and migration, and increases the release of cytoprotective factors. Together, these effects enhance the overall antifibrotic efficacy of MSCs in a models of liver fibrosis [149]. These results demonstrate that GDF15 substantially improves the therapeutic potential of MSCs by boosting their survival, migration, and secretory performance. Future studies should focus on elucidating the molecular mechanisms underlying the crosstalk between GDF15 and MSCs, as well as evaluating their clinical applicability, to facilitate the development of novel combination therapies for liver fibrosis.

## 5. Targeting GDF15 for the Treatment of Liver Fibrosis

GDF15 has emerged as a promising therapeutic target in liver fibrosis due to its direct regulatory effects on HSC activation and immune cell function. By simultaneously regulating these two central pathogenic processes, GDF15 functions as a key regulator of fibrogenesis. However, the expression of GDF15 follows a dynamic pattern during disease progression, which is typically downregulated in early liver injury but markedly upregulated in advanced fibrosis, highlighting the necessity for timed and context-dependent therapeutic strategies. Consequently, targeting GDF15 effectively requires stage-specific approaches that account for its shifting role from protective to pathogenic across the spectrum of liver disease.

### 5.1. Upregulation of GDF15 as a Strategy for Anti-Liver Fibrosis Therapy

Accumulating evidence establishes GDF15 as a critical hepatoprotective factor that mitigates the pathogenesis of liver fibrosis [223]. The functional importance of reduced GDF15 expression is evident in Gdf15-deficient mice, which develop exacerbated liver injury and fibrosis upon exposure to hepatotoxic agents. Conversely, therapeutic restoration of GDF15 signaling, achieved through either viral vector-mediated gene delivery or administration of recombinant protein, effectively attenuates fibrotic progression in experimental models. A primary mechanism through which GDF15 exerts its protective effects is by alleviating intrahepatic inflammation. GDF15 deficiency fosters a pro-inflammatory environment during fibrosis, characterized by enhanced recruitment of monocytes and neutrophils [97]. Crucially, GDF15 drives the functional reprogramming of macrophages toward an anti-inflammatory phenotype. The therapeutic relevance of this immunomodulation is confirmed by adoptive transfer experiments, wherein infusion of GDF15-conditioned macrophages into fibrotic mice significantly reduces inflammation and fibrosis [146]. In addition to these anti-inflammatory actions, GDF15 deficiency promotes profibrotic signaling, particularly through the TGF-β1/SMAD3 pathway, which is suppressed by exogenous GDF15 treatment [97]. The translational significance of these results is supported by human data, which show elevated circulating GDF15 levels in patients with metabolic liver diseases and advanced fibrosis, suggesting a compensatory, albeit insufficient, protective response [107,224]. Collectively, GDF15 serves as a pivotal endogenous safeguard against liver fibrosis by concurrently suppressing inflammatory responses and inhibiting key profibrotic signaling pathways, underscoring its potential as a novel therapeutic target.

**Figure 5:**
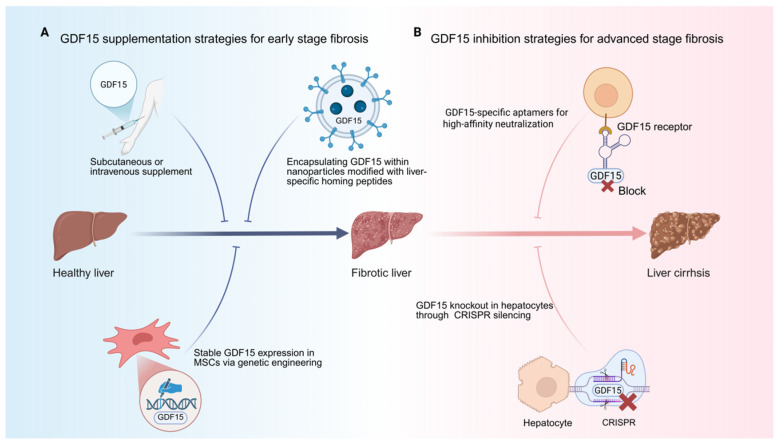
Stage-specific therapeutic strategies for targeting growth differentiation factor 15 (GDF15) in liver fibrosis. (**A**) GDF15 supplementation strategies for early-stage fibrosis. Based on the protective functions of GDF15 observed in preclinical studies, several therapeutic approaches have been proposed to restore its signaling in early liver fibrosis, where functional GDF15 may be deficient. Three primary strategies under investigation include: conventional administration of recombinant human GDF15 (rhGDF15); liver-targeted nanotherapy using peptide-functionalized nanoparticles for precise delivery; and engineered cellular therapy using genetically modified mesenchymal stem cells (MSCs) as a sustainable source of GDF15 production, which synergizes with their inherent tropism and intrinsic antifibrotic functions. While rhGDF15 has shown efficacy in preclinical models, the latter two approaches remain in early stages of development and warrant further validation in the context of liver fibrosis. (**B**) GDF15 inhibition strategies for advanced stage fibrosis. In advanced fibrosis, cirrhosis, and hepatocellular carcinoma (HCC), where pathological GDF15 overexpression has been implicated in disease progression, therapeutic strategies aimed at intercepting GDF15 signaling are being explored. These include durable intracellular CRISPR-mediated silencing, complemented by extracellular neutralization via high-affinity aptamers and GDNF family receptor alpha-like Fc (GFRAL-Fc) decoy receptors. Although neutralizing antibodies against GDF15 have demonstrated efficacy in preclinical cancer models, the application of these emerging approaches in advanced liver disease remains investigational and requires further study.

During the early antifibrotic phase (Figure 5A), supplementation with recombinant human GDF15 (rhGDF15) may be achieved through subcutaneous or intravenous administration. However, conventional exogenous GDF15 delivery faces significant challenges, including poor targeting specificity and rapid clearance. To overcome these limitations, two advanced targeted strategies offer promising solutions. One approach involves encapsulating GDF15 within nanoparticles modified with liver-specific homing peptides on their surface, thereby enhancing precise hepatic delivery and prolonging circulatory persistence. Alternatively, the intrinsic tropism of MSCs for HGF-enriched microenvironments can be exploited as a targeted delivery strategy. Genetic engineering can be used to achieve stable GDF15 expression in MSCs, thereby converting them into cellular carriers for localized and sustained GDF15 release. Accordingly, combining GDF15 modulation with MSC-based therapy represents a particularly promising therapeutic strategy. MSCs and their exosomes mitigate fibrosis through multiple mechanisms, including the delivery of antifibrotic circRNAs and miRNAs that induce HSC apoptosis or inhibit activation. GDF15 enhances MSC viability, migratory capacity, and paracrine function, thereby potentially amplifying their intrinsic antifibrotic effects [225,226].

Notably, advancing this therapeutic strategy necessitates addressing a key translational challenge. Systemic administration of GDF15 can activate brainstem GFRAL receptors, resulting in dose-limiting adverse effects such as nausea and emesis, as demonstrated in both clinical trials of GDF15 analogs and preclinical studies [227,228]. Critically, the proposed liver-targeted nanoparticle and engineered MSC-based approaches are designed precisely to confine GDF15 action to the liver, thereby maximizing local efficacy while minimizing systemic exposure and associated central side effects. The feasibility of these advanced delivery platforms is strongly supported by recent and analogous successes in liver fibrosis therapy. First, the therapeutic potential of targeted nanocarriers has been demonstrated using liver-homing lipid nanoparticles. In one study, these nanoparticles delivered mRNA encoding a therapeutic antibody, resulting in sustained local protein expression and superior reversal of steatohepatitis and fibrosis compared with systemic administration [10]. This provides direct proof-of-concept for using a similar nanoplatform to deliver GDF15. Secondly, the principle of engineering delivery vehicles for cellular precision is strongly supported by work showing that exosomes modified with a peptide targeting aHSC achieve specific accumulation in fibrotic tissue and enhanced anti-fibrotic efficacy [229], a strategy directly analogous to our proposed ligand-decorated systems. Finally, the potential and context-dependence of engineered MSC therapies are underscored by research highlighting that the host microenvironment, regulated by factors such as FSTL1 (follistatin-like protein 1), critically determines therapeutic outcome by modulating essential early immune cell recruitment [230]. This insight reinforces the rationale for engineering MSCs to express potent immunomodulators like GDF15, aiming to actively shape a favorable therapeutic microenvironment. Collectively, these independent lines of evidence substantiate the core concepts behind our proposed approaches, indicating that adapting these advanced delivery platforms for GDF15 could be a viable and promising strategy for anti-fibrotic therapy.

### 5.2. Anti-Liver Fibrosis by Inhibiting GDF15

Substantial clinical evidence reveals a paradigm shift for GDF15 from a hepatoprotective factor in early injury to a marker and probable driver of pathology in advanced liver disease. Circulating and hepatic GDF15 levels are markedly elevated with disease progression across etiologies [231]. In alcohol-associated liver disease, GDF15 is significantly upregulated in severe hepatitis, correlating with clinical severity scores such as model for end-stage liver disease [231]. Similarly, in metabolic dysfunction-associated steatotic liver disease, GDF15 concentrations are significantly higher in patients with steatohepatitis and advanced fibrosis compared to those with simple steatosis. Large-scale cohort studies, including analyses from the UK Biobank, establish GDF15 as a robust predictive biomarker for incident cirrhosis, with elevated levels detectable over a decade prior to clinical diagnosis [232]. Mechanistically, in established fibrosis, GDF15 may acquire direct profibrotic properties, such as promoting hepatic stellate cell activation [159]. Crucially, GDF15 emerges as a key mediator in the tumor microenvironment, where its secretion by HSC, dependent on their autophagic flux, significantly enhances HCC cell proliferation. This tumor-promoting effect is abolished by genetic deletion of GDF15 in stellate cells [161]. The clinical relevance is confirmed by the increased abundance of GDF15-positive cells in human HCC tissues and rising serum GDF15 levels with tumor progression, collectively positioning GDF15 as a significant contributor to the pathogenesis of late-stage liver disease.

Collectively, these findings highlight the context-dependent duality of GDF15 in liver disease. GDF15 may exert protective effects during the early stages of liver injury. However, in advanced disease, including cirrhosis and HCC, sustained GDF15 elevation appears to promote disease progression. This pathogenic effect is mediated by the establishment of a pro-tumorigenic microenvironment and the activation of fibrotic and oncogenic signaling pathways. Given the established pathogenic role of GDF15, targeting its signaling represents a rational strategy for advanced disease (Figure 5B). In addition to conventional methods, novel interventions are being developed to inhibit this pathway with precision. Intracellularly, durable suppression can be achieved through CRISPR-based transcriptional silencing. Extracellularly, GDF15-specific aptamers provide high-affinity neutralization, while soluble GFRAL-Fc decoy receptors sequester the circulating ligand, working in concert to block the initiation of signaling.

**Figure 6:**
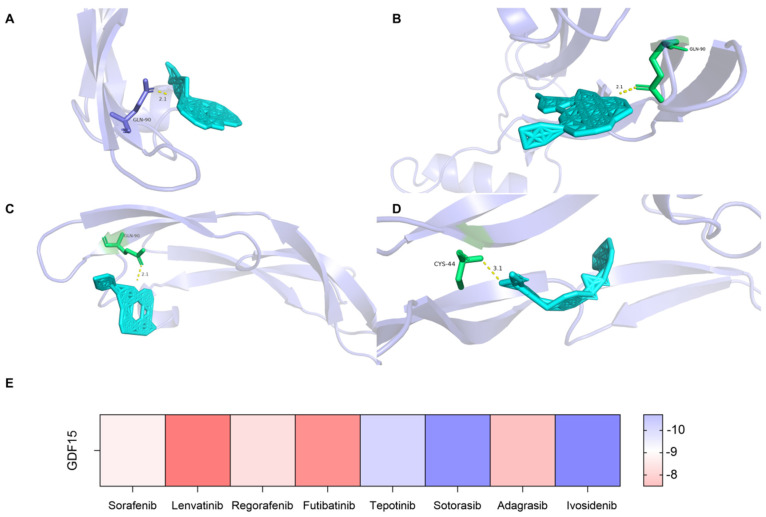
Molecular docking analysis of small-molecule inhibitors with growth differentiation factor 15 (GDF15) (PDB:Q99988). The schematic is based on a computationally predicted model and does not represent an experimentally validated structure; the docking analysis is presented as a hypothesis-generating, illustrative approach only. (**A**) Predicted binding pose of Sotorasib docked to the GDF15 region (residues 197–308); Sotorasib is predicted to localize near residue 286. (**B**) Predicted binding pose of Ivosidenib, also near residue 286. (**C**) Predicted binding pose of Tepotinib, near residue 286. (**D**) Predicted binding pose of Sorafenib, predicted to dock near residue 240. (**E**) Heatmap depicting calculated binding affinities (ΔG, kcal/mol) for eight kinase inhibitors: Sotorasib (−10.7), Ivosidenib (−10.0), Tepotinib (−9.7), Sorafenib (−9.0), Regorafenib (−8.9), Adagrasib (−8.7), Lenvatinib (−7.9), and Futibatinib (−7.5). Additionally, docking positions for Lenvatinib (residues 242/244), Regorafenib (242), Futibatinib (246), and Adagrasib (244) are localized within key regions of the GDF15 functional domain.

In addition to direct targeting strategies, we explored a hypothesis-generating approach aimed at assessing whether existing clinical-stage drugs might theoretically interact with GDF15. We postulated that some compounds, although not originally designed to target secreted cytokines, could in principle engage structural regions of GDF15 and thereby warrant further experimental investigation. To preliminarily explore this possibility, we performed a structure-based molecular docking analysis using a computationally predicted AlphaFold model of GDF15, screening eight clinical-stage HCC drugs against a region previously implicated in receptor engagement (residues 197–308) (Figure 6). Importantly, this analysis was conducted solely as an exploratory in silico exercise, and the predicted interactions should not be interpreted as evidence of direct binding or functional inhibition. The docking analysis suggested that several compounds could be positioned near residues involved in GFRAL engagement. First, all tested compounds were predicted to dock in proximity to regions associated with GDF15–receptor interaction, with several drugs exhibiting relatively higher predicted affinities. Second, the predicted binding poses clustered predominantly within residues 240–286. For example, Sorafenib and Lenvatinib were predicted to interact near residues 240–244, whereas Sotorasib and Ivosidenib showed predicted binding near residue 286. However, given that these agents were developed to target intracellular kinases and that GDF15 is a secreted protein, these findings remain theoretical and require rigorous biochemical and functional validation. Collectively, this analysis serves to generate testable hypotheses rather than to establish a novel mechanism of action.

Although clinical trials directly targeting GDF15 for liver fibrosis are currently lacking, translational experiences from oncology have demonstrated the clinical feasibility of pharmacologically modulating the GDF15 pathway in humans. Several GDF15-targeted agents, including visugromab, ponsegromab, and AZD8853, have entered early-phase clinical trials for advanced cancers and cancer-associated cachexia, providing valuable insights into therapeutic targeting strategies, safety profiles, and pharmacodynamic monitoring of GDF15 modulation (Table 2). However, given the context-dependent functions of GDF15, particularly its potential hepatoprotective and antifibrotic effects in chronic liver disease, future therapeutic approaches for liver fibrosis may require precise modulation of GDF15 activity rather than direct extrapolation of GDF15 inhibition strategies developed in oncology.

### 5.3. Resolving the Dual Nature of GDF15: A Unifying Framework

To further clarify the conceptual landscape of GDF15 function in liver fibrosis, we propose a multi-dimensional framework that distinguishes four key contextual axes: disease stage (early versus advanced fibrosis), pathological milieu (inflammatory versus tumor-bearing), signaling mode (GFRAL-dependent central versus GFRAL-independent peripheral), and evidence strength (robust versus preliminary). This framework serves as a guide for interpreting the experimental data and for designing future studies.

A stage- and context-dependent model reconciles the apparent paradox of GDF15 in liver fibrosis (Table 3 and Table 4). In early injury, GDF15 functions as a protective stress response. Its deficiency exacerbates fibrosis by derepressing TGF-β1/SMAD3 signaling [97,146], whereas exogenous GDF15 suppresses HSC activation and inflammation [145,150]. Nuclear pro-GDF15 further antagonizes SMAD-dependent transcription [130]. In advanced fibrosis or HCC, chronic GDF15 elevation becomes maladaptive. Antibody-mediated neutralization attenuates fibrosis [159], and GDF15 promotes HSC proliferation via ERK/SMAD3 under hypoxia [160], while HSC-derived GDF15 drives tumor growth [161]. The net effect also varies by cellular source. Hepatocyte-derived GDF15 is protective [150,175], whereas HSC-derived GDF15 promotes pathology [161]. Microenvironmental cues, including hypoxia and the TGF-β-rich milieu, further modulate GDF15 activity by biasing signaling toward pro-fibrotic outcomes [160].

An assessment of the evidence supports the following: The anti-fibrotic role of GDF15 is most robustly supported by knockout studies, where deficiency exacerbates fibrosis via TGF-β/SMAD3 de-repression and inflammation [97,146]. Hepatocyte-derived protection in alcohol-induced injury is well validated [175], and anti-inflammatory effects on macrophages and T cells are confirmed [146,150]. Direct anti-fibrotic effects on HSC remain preliminary, based on in vitro pharmacological doses [145] without conditional knockout confirmation.

The strongest pro-fibrotic and pro-tumorigenic evidence comes from HCC-HSC crosstalk, where HSC-derived GDF15 promotes tumor growth via autophagy-dependent secretion [161]. ERK/SMAD3 activation in hypoxic HSC is supported in vitro and in vivo, though the receptor is unknown [160]. GDF15 neutralization attenuates fibrosis in CCl_4_ and TAA models [159], but downstream mechanisms are less defined.

The central GFRAL-dependent axis is well established [19,20,105]. Peripheral hepatic actions, including AMPK activation, gluconeogenesis suppression [97], and immunomodulation [146,150], have been demonstrated in whole-animal or in vitro systems, but their mechanisms remain uncertain. Whether these effects involve GFRAL, TGF-βRI/II [118], or nuclear pro-GDF15 [130] remains to be resolved using cell-type-specific receptor knockout models.

Collectively, this reconciled model supports stage-specific therapeutic strategies. GDF15 augmentation is appropriate for early fibrosis, whereas GDF15 inhibition is preferable in advanced disease or HCC. Tissue-targeted delivery remains essential to minimize central side effects, particularly nausea and emesis associated with GFRAL activation [227,228].

## 6. Future Perspectives and Outstanding Questions

### 6.1. Unresolved Mechanistic Questions

Several critical questions regarding GDF15 in liver fibrosis remain unresolved. Chief among these are the receptor and signaling switches that dictate protective versus pathogenic outcomes. Specifically, we need to understand the context-dependent transition from TGF-β/SMAD3 inhibition to ERK/SMAD3 activation. The distinct roles of nuclear pro-GDF15 and secreted mature GDF15 also require clarification. Global knockout models obscure cell-type-specific contributions. Conditional knockouts combined with single-cell RNA sequencing and spatial transcriptomics are essential to dissect cellular sources and targets. Most mechanistic insights derive from a limited number of studies. Therefore, independent validation across diverse fibrosis models and human-relevant systems such as organoids and precision-cut liver slices is urgently needed. Proposals such as engineering GDF15-secreting B cells and extrapolating findings from COVID-19 ARDS to liver fibrosis remain theoretically interesting but unproven. They require rigorous liver-specific preclinical testing. The reconciled model supports stage-specific strategies. However, practical challenges persist. These include identifying reliable stratification biomarkers and developing liver-targeted delivery to minimize systemic side effects [227,228].

### 6.2. Therapeutic Strategies and Safety Considerations

The context-dependent duality of GDF15 necessitates matching therapeutic intervention to disease stage. Physiological plasma GDF15 averages ~450 pg/mL in humans and ~100 pg/mL in mice [236]. It increases with age, reaching over 2100 pg/mL in octogenarians [236]. In malignancy, levels can reach 10,000–100,000 pg/mL, and approximately 19,000 pg/mL during pregnancy [236]. By contrast, the anorectic and aversive effects of GDF15 require pharmacological concentrations exceeding 100 ng/mL [104,237]. This is orders of magnitude above the physiological range. Endogenous elevation, such as that induced by prolonged exercise, does not suppress appetite [104,237]. Combined deletion of Gdf15 and Fgf21 minimally affects metabolism [237]. Pharmacological dosing with long-acting GDF15, however, induces significant weight loss [237]. Most preclinical anti-fibrotic studies have used supraphysiological recombinant GDF15 [145]. It remains uncertain whether protective effects are achievable at clinically relevant lower concentrations. This underscores the need for dose–response studies to define a therapeutic window [237].

GDF15 agonism would be desirable in early-stage fibrosis but raises considerable safety concerns. Central GFRAL-RET activation can trigger anorexia, nausea, and cachexia [104,238]. These effects are especially hazardous in malnourished patients [23,24]. Nausea and emesis also represent dose-limiting toxicities [227,228]. Systemic agonism in patients with occult HCC could theoretically promote tumor growth [160,161]. This mandates rigorous HCC screening and highlights the necessity of liver-restricted delivery. Conversely, GDF15 antagonism is preferable in advanced fibrosis or HCC. In these settings, sustained GDF15 elevation drives fibrogenic and tumorigenic signaling. Neutralizing antibodies reverse weight loss and preserve lean mass in tumor models [239]. GFRAL blockade maintains mass under calorie restriction [240]. Ponsegromab has advanced to Phase 3 trials for cancer cachexia [238,241]. Antagonism therefore represents the more clinically mature approach.

Risks of GDF15 antagonism also require careful evaluation. Cardioprotective concerns arise because GDF15 blockade prevented cachexia in a heart failure model. However, it may disrupt compensatory pathways [242]. Human cardiac effects warrant further study. Host defense data are conflicting: GDF15 deficiency can enhance pathogen clearance in some models yet exacerbate tissue injury in others [104,238]. Human loss-of-function carriers lack overt metabolic phenotypes [104]. However, the long-term consequences of chronic antagonism in liver disease remain unknown. In summary, safe clinical translation will require rigorous safety studies and patient stratification based on disease stage, nutritional status, tumor surveillance, and GDF15 expression.

## 7. Discussion

GDF15 plays a context-dependent dual role in liver fibrosis, exerting protective effects during early-stage injury while potentially contributing to disease progression in advanced stages. Its core mechanism involves the simultaneous regulation of HSC activation and immune microenvironment homeostasis, establishing GDF15 as a key and multifaceted therapeutic target. Supporting evidence from animal models suggests its regulatory function, and clinical studies have reported correlations between GDF15 expression levels and disease severity in patients. However, the causal relationship between GDF15 elevation and disease progression in humans requires further investigation [107]. Nevertheless, the molecular mechanisms governing its functional transition from protective to pathogenic remain poorly defined. A more detailed understanding of its crosstalk with other key signaling pathways, such as TGF-β and Wnt/β-catenin, in various cell types, including hepatocytes and Kupffer cells, is still required.

The reconciled framework presented in Section 5.3 (Table 1) provides a conceptual basis for understanding this duality, but several critical questions remain unresolved. Future studies should focus on advanced methodologies such as conditional knockout mouse models, with GDF15 deletion in hepatocytes or HSC, to clarify its cell-type-specific functions. Techniques like single-cell RNA sequencing should be employed to map the transcriptional landscape of diverse liver cell populations in response to GDF15 signaling. This approach may reveal novel mechanistic insights. Furthermore, the potential of GDF15 as a biomarker, either alone or in combination with established clinical tools such as the enhanced liver fibrosis (ELF) test, for predicting fibrosis progression and treatment response requires rigorous validation.

The development of GDF15-targeted therapies should be tailored to the stage of liver disease. During early fibrosis, strategies aimed at enhancing GDF15 signaling, such as long-acting recombinant proteins or small-molecule agonists, may potentiate its beneficial anti-inflammatory and antifibrotic effects. Conversely, in advanced fibrosis or HCC, intervention should focus on inhibiting GDF15 using neutralizing antibodies, soluble receptor traps, or RNA-based strategies to counteract its detrimental roles. Combining GDF15-targeted agents with existing therapies, such as anti-inflammatory or antiviral drugs, or with other investigational agents, represents a promising direction for achieving synergistic efficacy. While computational modeling provides useful structural hypotheses, definitive conclusions regarding GDF15–drug interactions will require direct biochemical and functional validation. Importantly, achieving tissue-specific delivery of these interventions will be critical for minimizing off-target effects and ensuring treatment safety.

## 8. Conclusions

In conclusion, GDF15 represents a critical biological interface linking fibrotic and inflammatory pathways, establishing it as a compelling target for precision medicine in liver fibrosis. Although significant challenges remain in fully elucidating its context-dependent mechanisms, continued research and the advancement of stage-specific therapeutics offer considerable potential for enhancing clinical outcomes in this disease.

## Figures and Tables

**Table 1:** Overview of GDF15 Functional Regulation, Biological Effects, and Its Biomarker and Therapeutic Potential Across Different Cell Types.

Cell Type	Representative Intervention	Effect on GDF15	Biological Effect	Biomarker Implication
HSC	Recombinant GDF15 protein treatment (in vitro)	Increased	Anti-fibrotic: Exogenous GDF15 directly reduces fibrotic gene and OPN expression;	Elevated serum/hepatic GDF15 levels may serve as a potential diagnostic/monitoring biomarker for NASH and related liver fibrosis. GDF15 is a potential diagnostic, staging, and prognostic biomarker for HCC
	Gdf15 gene knockout (in vivo)	Decreased	Endogenous GDF15 deficiency exacerbates HSC activation and liver fibrosis.	
	Recombinant human GDF15 (exogenous)	Increased	Promotes proliferation, cell cycle progression, ECM gene upregulation, activates ERK/SMAD3	
	Atg7 gene knockout	Decreased	Inhibits HSC activation;	
	GDF15 gene knockout	Decreased	Suppresses tumor growth and proliferation	
	GDF15 gene overexpression	Increased	No effect on HSC activation; Promotes tumor growth and ERK/AKT signaling	
Macrophages	Recombinant rmGDF15 treatment	Increase	Metabolic reprogramming (enhanced OXPHOS), anti-inflammatory phenotype, suppressed M1	GDF15 as an immunometabolic modulator. Cell therapy potential, reflecting GDF15 efficacy. Suggests targeting this axis for anti-inflammation therapy, not merely a biomarker. Plasma GDF-15 reflects ATM infiltration in obesity, indirectly indicating insulin resistance and T2D risk
	Transfer of GDF15-preprogrammed macrophages	Cells carry GDF15 function	Reduced CCl_4_-induced liver injury and fibrosis	
	GDF15 + ADRB2 agonist (CBL) treatment in vitro	Exogenous addition (Gain-of-function)	Activates PKA pathway, induces Caspase-9/3-dependent apoptosis, lowers Bcl-2, suppresses inflammatory cytokines	
	GeRPs-delivered siGdf15 silencing in Adipose tissue macrophages (ATMs)	Decreased	Increased body weight gain	
T Cell	Recombinant GDF15 addition	Increased	Induced iTreg differentiation; enhanced nTreg suppressive function; mechanistically stabilizes FOXP3 via CD48-ERK-STUB1 axis.	Low or absent GDF15 suggests insufficient protective factor and increased disease susceptibility. Reveals a novel anti-inflammatory mechanism of GDF15: enhancing Treg suppressive function to maintain immune homeostasis.
	GDF15 knockout	Loss of function	Exacerbates alcoholic steatosis and CCl_4_ fibrosis; increases intrahepatic activated CD4^+^/CD8^+^ T cells and TNF-α^+^CD8^+^ T cells	
	Direct recombinant GDF15 (rGDF15) treatment	Increase	No direct effect on CD4^+^/CD8^+^ T-cell activation, Th17 differentiation, or cellular energy metabolism (OCR/ECAR).	
	rGDF15 treatment + co-culture	Increase	Enhances Treg-mediated suppression of effector T-cell proliferation and IFN-γ production via IL-10 upregulation; no effect on senescent CD8^+^ T cells.	
NK cell	Anti-GDF-15 antibody depletion from serum	Decreased	Reduced SMAD1/5 activation; direct restoration of NK function not confirmed	Suggests therapeutic targeting of this pathway. Confirms direct suppressive effect on NK cells.
	TGF-βRI inhibitor	Unchanged (signaling blockade only)	Partially restored IL-12Rβ2 expression and IFN-γ production	
	Recombinant human GDF-15 (10 ng/mL)	Increased	Downregulated IL-12Rβ2 expression in some donors	
Hepatic B cells (from fibrotic mice)	Only transcriptomic detection, no intervention	Increased	Not investigated	Not mentioned
B-CLL cells (CD19^+^)	Nutlin-3 (MDM2 inhibitor)	Increased	Not investigated	Not mentioned
Mouse bone marrow-derived MSCs	Recombinant GDF15 (50 ng/mL, 24 h) pretreatment	Exogenous GDF15 increases total level; endogenous GDF15 rises at 24 h but falls at 48 h under hypoxia	Anti-apoptosis, ROS reduction, improved mitochondrial function, enhanced paracrine effects; in vivo: increased survival, improved cardiac function, reduced fibrosis	GDF15 may indicate ischemia/oxidative stress; a sustained decline may reflect depletion of protective reserves (requires clinical validation; not supported as an independent biomarker by this study)

**Table 2:** Clinical Trial Overview of Anti-GDF15 Monoclonal Antibodies in Cancer.

Target	Mechanism	Phase	Disease	Outcome	Implication	Trial	Ref
Ponsegromab	Humanized monoclonal antibody inhibiting GDF15 signaling	II	Cancer cachexia with elevated serum GDF15	Increased body weight, improved cachexia symptoms and physical activity, with safety comparable to placebo	Validates GDF15 as a therapeutic target and supports clinical feasibility of GDF15 inhibition	NCT05546476	[233]
Ponsegromab	Neutralizes circulating GDF15 and blocks GDF15–GFRAL signaling	Ib	Cancer cachexia	Reduced unbound serum GDF15; increased body weight (~5.2 kg vs. historical control), improved appetite/activity, and showed acceptable tolerability	Demonstrates feasibility of systemic GDF15 modulation and pharmacodynamic biomarker monitoring	NCT05546476	[234]
AZD8853	Neutralizes GDF15 and reduces circulating free GDF15	I/IIa	Advanced solid tumors	Well tolerated but showed no measurable antitumor activity; transient GDF15 suppression	Highlights challenges in achieving sustained GDF15 inhibition	Not reported	[235]
Visugromab	Neutralization of GDF15	I/IIa	Anti-PD-1/PD-L1 refractory advanced tumors	Well tolerated; induced objective tumor regressions and CRs in subsets of patients	Demonstrates feasibility of clinical GDF15 targeting and provides experience in pharmacological modulation and biomarker assessment	NCT04725474	[116]

**Table 3:** Anti-fibrotic effects of GDF15 in experimental models of liver fibrosis.

Model/Context	Species/Cells	Genetic/Pharmacological Manipulation	Effect on Fibrosis	Mechanisms	Ref.
CCl_4_- induced fibrosis	Mouse	GDF15 knockout	Exacerbates fibrosis	↑ TGF-β1/SMAD3 signaling; ↑ α-SMA expression; ↑ profibrotic gene expression	[97]
DDC- induced fibrosis	Mouse	GDF15 knockout	Exacerbates fibrosis	↑ HSC activation; exacerbate the inflammatory microenvironment	[146]
Dietary MASH model	Mouse; Primary HSC	GDF15 knockout; rGDF15 treatment	Anti-fibrotic	↓ Tgfb1, Col1a1, Timp1, Acta2, osteopontin; direct inhibition of HSC profibrogenic genes	[145]
Alcohol-induced liver injury	Mouse; Kupffer cells	Hepatocyte-derived GDF15	Anti-inflammatory/protective	↑ ADRB2 expression on KCs → KC apoptosis; ↓ NF-κB activation	[175]
CCl_4_-induced fibrosis	Mouse	GDF15 (via Nrf2-ARE pathway)	Anti-fibrotic	↑ antioxidant enzyme expression; ↓ oxidative stress and inflammation	[129]
CCl_4_-induced fibrosis	Mouse	GDF15 modulation of T cells	Anti-fibrotic	↓ CD4^+^/CD44^+^ and CD8^+^/CD44^+^ pro-inflammatory T cells; ↓ NF-κB, JNK, p38 signaling; ↑ naïve T-cell populations	[150]

Symbols: ↑, upregulation/increase; ↓, downregulation/decrease; →, induction/leading to. CCl4, Carbon Tetrachloride.

**Table 4:** Pro-fibrotic and pro-tumorigenic effects of GDF15 in experimental models of liver fibrosis.

Model/Context	Species/Cells	Genetic/Pharmacological Manipulation	Effect on Fibrosis	Putative Mechanisms	Ref.
CCl_4_-induced fibrosis	Mouse	Anti-GDF15 neutralizing antibody	Anti-fibrotic (Neutralization attenuates)	↓ TGF-β-mediated HSC activation; ↓ fibrosis severity	[159]
TAA-induced fibrosis	Mouse	Anti-GDF15 neutralizing antibody	Anti-fibrotic (Neutralization attenuates)	↓ TGF-β-mediated HSC activation; ↓ fibrosis severity	[159]
Hypoxia/Chemotherapy	HCC cells; HSC	GDF15 overexpression; GDF15 neutralization	Pro-fibrotic	↑ MAPK pathway in HCC → ↑ HSC proliferation and collagen production via ERK1/2 and SMAD3 activation	[160]
HCC-HSC crosstalk	Human/Mouse HSC; HCC cells	Autophagy inhibition; GDF15 knockdown	Pro-fibrotic/Pro-tumor	HSC autophagy → GDF15 secretion → ↑ HCC proliferation; reciprocal crosstalk promotes ECM deposition	[161]
Systemic inflammation	NK cells	GDF15 via TGF-βRI	Pro-fibrotic (indirect)	↑ SMAD1/5/8 → ↓ IL-12Rβ → ↓ NK cell IFN-γ production → ↓ antifibrotic NK activity	[202]

Symbols: ↑, upregulation/increase; ↓, downregulation/decrease; →, induction/leading to. CCl4, Carbon Tetrachloride.

## Data Availability

No new data were created or analyzed in this study. Data sharing is not applicable.

## Abbreviations

The following abbreviations are used in this manuscript:

ECM	extracellular matrix
HSCs	hepatic stellate cells
HCC	hepatocellular carcinoma
GDF15	growth differentiation factor 15
MIC-1	macrophage inhibitory cytokine 1
NAG-1	NSAID-Activated Gene 1 Protein
PTGFB	placental TGF-Beta
TGF-β	transforming growth factor-β
GFRAL	glial cell line-derived neurotrophic factor receptor alpha-like
GDNF	glial cell line-derived neurotrophic factor
ERK	extracellular signal-regulated kinase
HBV	hepatitis B
HCV	hepatitis C
WHO	World Health Organization
DAMPs	damage-associated molecular patterns
JNK	c-Jun N-terminal kinase
SATB1	AT-rich sequence-binding protein 1
IL-6	interleukin-6
ROS	reactive oxygen species
ICAM-1	intercellular adhesion molecule-1
ALD	Alcohol-related liver disease
MASH	metabolic dysfunction-associated steatohepatitis
NLRP3	NOD-like receptor family, pyrin domain-containing 3
NAFLD	Non-alcoholic fatty liver disease
ER	endoplasmic reticulum
NF-κB	nuclear factor kappa-light-chain-enhancer of activated B cells
SREBP-1c	sterol regulatory element-binding protein 1c
SREBP-2	sterol regulatory element-binding protein 2
VLDL	very low-density lipoprotein
SMAD	sma- and mad-related proteins
Wnt	wingless-related integration site
JAK	β-catenin, and janus kinase
STAT	signal transducer and activator of transcription
α-SMA	α-smooth muscle actin
Col-I	type I collagen
Col-III	type III collagen
TGF-β1	transforming growth factor-β1
TβRII	TGF-β receptor II
TβRI	TGF-β receptor I
MMP1	matrix metalloproteinase-1
LOXL1	lysyl oxidase-like 1
TIMP-1	tissue inhibitor of metalloproteinase-1
PAMPs	pathogen-associated molecular patterns
TNF-α	tumor necrosis factor-alpha
CCL2	C-C motif chemokine ligand 2
CXCL1	C-X-C motif chemokine ligand 1
UPR	unfolded protein response
AP	area postrema
NTS	tractus solitarius
PLCγ	phospholipase C gamma
AIH	autoimmune hepatitis
rGDF15	recombinant GDF15
Nrf2-ARE	Nrf2-antioxidant response element
NK	natural killer
MSCs	mesenchymal stem cells
MAPK	mitogen-activated protein kinase
AMPK	adenosine monophosphate-activated protein kinase
mTOR	mechanistic target of rapamycin pathway
AGEs	advanced glycation end-products
HIF-1α	hypoxia-inducible factor-1α
Drp1	dynamin-related protein 1
OXPHOS	oxidative phosphorylation
ETC	mitochondrial electron transport chain
mtUPR	mitochondrial unfolded protein response
CCl_4_	carbon tetrachloride
TAA	thioacetamide
M1 macrophages	classically activated macrophages
M2 macrophages	alternatively activated macrophages
VEGF	vascular endothelial growth factor
HGF	hepatocyte growth factor
Areg	amphiregulin
Trm	CD8^+^ tissue-resident memory T cells
CCR5	C-C chemokine receptor type 5
FasL	Fas ligand
FOXP3	fork head box P3
iTreg	induced Treg
nTreg	natural Treg
NKG2D	natural killer group 2 member D
NKp46	natural killer cell p46-related protein
BAX	BCL2-associated X protein
EP3	prostaglandin E2 receptor 3
PKC	protein Kinase C
VCAM1	vascular cell adhesion molecule 1
TGF-βRI	TGF-β receptor I
EGF	epidermal growth factor
NO	nitric oxide
PGE2	prostaglandin E2
IDO	indoleamine 2,3-dioxygenase
HLA-G5	human leukocyte antigen G isoform 5
MDSCs	myeloid-derived suppressor cells
qHSC	quiescent HSC
aHSC	activation of HSC
rhGDF15	recombinant human GDF15
